# Post-translational modification gene signatures implicate FBXW7 in immune and vascular dysregulation of Moyamoya disease

**DOI:** 10.3389/fgene.2025.1723233

**Published:** 2025-12-18

**Authors:** Xiaofan Yu, Zicong Wang, Zhenyu Zhou, Yutong Liu, Shihao He, Yuanli Zhao

**Affiliations:** 1 Department of Neurosurgery, Beijing Chao-Yang Hospital, Capital Medical University, Beijing, China; 2 Department of Neurosurgery, Peking Union Medical College Hospital, Peking Union Medical College and Chinese Academy of Medical Sciences, Beijing, China

**Keywords:** Fbxw7, immune, Moyamoya disease, post-translational modifications, vascular dysregulation

## Abstract

**Background:**

Moyamoya disease (MMD) is a rare cerebrovascular disorder characterized by progressive stenosis and occlusion of intracranial arteries and abnormal collateral vessel formation. However, the underlying molecular mechanisms are not completely understood. Post-translational modifications (PTMs) are essential regulators of protein function and signal pathway, yet their roles in MMD have not been fully explored.

**Methods:**

Transcriptomic datasets from an independent cohort and public datasets were integrated. Differential expression analysis, weighted gene co-expression network analysis (WGCNA), and machine learning were applied to identify candidate biomarkers. Functional enrichment, immune infiltration analysis, and protein-protein interaction networks were constructed. Validation of expression and diagnostic performance of the selected feature genes was performed across training and test sets. Molecular docking and dynamics simulations were conducted to evaluate the potential drug molecules. In addition, enzyme-linked immunosorbent assay (ELISA) was performed to measure serum FBXW7 levels in an independent cohort (n = 20), and *in vitro* experiments including scratch assay and EdU assays were conducted to assess vascular smooth muscle cell (HBVSMC) migration and proliferation after FBXW7 knockdown.

**Results:**

1,547 differentially expressed genes were identified, and 4 genes were identified as feature genes with diagnostic significance, using machine learning and validation. With functional annotations, pathways including ubiquitination, SUMOylation, and neddylation were identified. HLA-A was upregulated and strongly associated with immune infiltration. FBXW7 was downregulated and may promote aberration in vascular smooth muscle cell proliferation and migration. Furthermore, molecular docking and dynamic simulations jointly confirmed the stability of the FBXW7-MRK-003 complex. ELISA results showed that serum FBXW7 levels were significantly decreased in MMD patients compared with healthy controls. Consistently, *in vitro* assays demonstrated that HBVSMC migration and proliferation were markedly enhanced in the si-FBXW7 group compared with the si-NC group.

**Conclusion:**

Our study identifies PTM-related genes involving the development of MMD. Especially, we found that FBXW7 and HLA-A bridging immune dysregulation and vascular dysfunction. Our work provide innovative insights into mechanisms of MMD pathogenesis and candidate therapeutic targets for precision therapy of MMD.

## 1 Introduction

Moyamoya disease (MMD) is a type of chronically progressive cerebrovascular disease, characterized by progressive stenosis or occlusion of the terminal internal carotid artery and the initial segments of the anterior and middle cerebral arteries, accompanied by the formation of compensatory collateral blood vessels (Scott and Smith, 2009). The clinical manifestations of MMD are complicated and diverse, including ischemic or hemorrhagic strokes. To date, no pharmacological treatment has been validated to successfully reverse the pathophysiological progression of MMD. Revascularization surgery is the primary treatment for MMD since it ameliorates cerebral hemodynamics (He et al., 2025a). However, surgeries carry risks including postoperative hyperperfusion, re-hemorrhage, ischemic stroke, and temporary neurological dysfunction (Mukerji et al., 2015; Kim et al., 2024; Tokairin et al., 2018; Lim et al., 2024; Kuroda and Houkin, 2008). Histopathological examinations illustrate intimal hyperplasia in affected vessels, with proliferation of smooth muscle cells and irregular undulation of the internal elastic lamina (Li et al., 1991).

Currently, there is plenty of research working on the mechanisms of MMD. Literature supports that genetic susceptibility may play a role in the development of MMD. Notably, The RNF213 gene, which is located in the q25.3 region of human chromosome 17, indicates a strong correlation with MMD. And the RNF213 p.R4810K mutations are more generally found in East Asian than Caucasian patients (Fang et al., 2024). Liu et al. recently reported that necroptosis and necroinflammation contributed to the pathogenesis of MMD under the dysfunction of immune response (Liu et al., 2025). And our previous work also suggested that immune system contributes to the pathogenesis of MMD by affecting the angiogenesis to promote endothelial thickening, which is accompanied by abnormalities in actin and cytoskeleton (Zhou et al., 2025). In addition, epigenetics including methylation profiles have been suggested to play a regulatory role in the development of MMD, especially in endothelial cell and immune cell interactions (Xu et al., 2024; Tokairin et al., 2025; He et al., 2025b). However, most studies focused on the transcriptome level or single-gene mutations. The role of regulatory mechanisms at protein levels, especially post-translational modifications (PTMs), remains largely unexplored.

Various characters of proteins are dynamically regulated by PTMs, including protein activity, localization, stability, and interaction with other proteins (Wang et al., 2022). PTMs extensively involve signal transduction, gene expression, and immune responses, thereby significantly regulating the functions of vascular endothelial cells, smooth muscle cells, and immune cells (Shen et al., 2020; Shen et al., 2024; Li et al., 2021). For instance, in the human Umbilical vein endothelial cells (HUVEC), STAT3 phosphorylation was promoted under hypoxia, and then upregulated vascular endothelial growth factor (VEGF) and angiopoietin 2 (ANGPT2) to regulate the proliferation, migration, and tube formation of HUVEC (Jia et al., 2022). In the vascular diseases, PTMs of VEGFR2 play a vital role in pathogenesis. It has been illustrated that ubiquitinaion regulates the stability and signal transduction of VEGFR2 while acetylation promotes conformational changes of VEGFR2 and thus positive regulates the transduction. Recent evidence has also highlighted the potential involvement of the biquitin-related genes in the pathogenesis of Moyamoya disease. In our previous work, we identified several ubiquitin-related genes, including ANAPC11, UCHL1, and USP41, which were significantly dysregulated in patients with Moyamoya disease and were closely associated with vascular remodeling and immune regulation. These findings suggest that abnormal ubiquitination may contribute to endothelial dysfunction and smooth muscle proliferation in Moyamoya disease. Building upon this, the present study extends the investigation from ubiquitin-related pathways to a broader post-translational modification (PTM) network, aiming to clarify how PTM-associated mechanisms collectively shape immune and vascular homeostasis in Moyamoya disease (Niu et al., 2025). Notably, SUMOylation of VEGFR2 suppress angiogenesis by affecting the subcellular localization of VEGFR2 (Singh et al., 2007; Zecchin et al., 2014). And the SUMOylation is upregulated under diabetic conditions which contributes to the impaired angiogenesis in diabetes (Zhou et al., 2018). PTMs dynamically regulate the physiological and pathological processes in the vascular system which may underlie the unexplored mechanisms of MMD.

In this study, we integrated transcriptomic analyses results of MMD and control samples and identified several PTM-related differentially expressed genes (DEGs) related to MMD. Machine learning was utilized to select 4 feature genes with great diagnostic significance. These genes demonstrated consistent dysregulation of immune filtration which may contribute to the development of MMD. Additionally, molecular docking and dynamics simulations were generated to predict potential drug targets based on the 4 feature genes. This study provides novel insights into PTM-related mechanisms in MMD and innovative idea for potential biomarkers and therapeutic targets.

## 2 Methods

### 2.1 Data resource and study participants

Gene expression data and sample information of moyamoya disease transcriptome dataset GSE189993 and GSE141024 were downloaded from GEO (Barrett et al., 2013) database (https://www.ncbi.nlm.nih.gov/geo/). The GSE189993 and GSE141024 dataset were combined to form a training set, while an independently generated dataset was used as the validation test.

The 20 post-translational modifications (PTM) gene sets were mainly obtained from literature PMID40010763 (Zhang et al., 2025). Among these, lactylation-related gene sets were acquired from literature PMID 37242427 (Cheng et al., 2023), while ubiquitination, sumoylation, neddylation, and deubiquitination-related gene sets were downloaded from MSigDb dataset as corresponding pathway gene sets (https://www.gsea-msigdb.org/gsea/msigdb/index.jsp). Detailed sources are provided in Table 2.

Patients examined between January 2020 and August 2025 with MMD were clinically diagnosed by medical history and neuroradiological examination, including cerebral digital subtraction angiography, magnetic resonance imaging, and functional regional cerebral blood flow studies following the diagnostic guidelines.

Peripheral blood samples were collected from all patients by venipuncture using a CPT Vacutainer containing sodium citrate as an anticoagulant (BD Biosciences, Heidelberg, Germany) prior to revascularization surgery. Blood samples were collected from healthy adult controls. The Vacutainers were centrifuged for 8 min at 460 g. The serum with lymphocytes was transferred into a new Falcon tube (352096, Corning) and centrifuged again at 3000 r/min for 15 min. The serum supernatant from ten patients with Moyamoya disease and ten age and sex matched healthy controls was collected, transferred into sterile cryovials, and stored at 80 °C prior to use. Healthy controls were recruited from the same hospital. All individuals in the healthy group were age and sex matched to the patients. Each healthy volunteer underwent a review of medical history and routine clinical evaluation at enrollment to confirm eligibility. This included no history of hypertension, diabetes, hyperlipidemia, coronary heart disease, autoimmune disorders, chronic inflammatory diseases, or any form of cerebrovascular disease (Supplementary Table S3).

Samples of the superficial temporal artery (STA) with a size of approximately 1–3 mm were obtained from ten patients with Moyamoya disease and three patients with intracranial aneurysm. These blood vessel specimens were immediately shock frozen in liquid nitrogen prior to RNA extraction (Supplementary Table S4).

### 2.2 Data preprocessing

The R package ArrayExpress (Version 1.62.0, http://www.bioconductor.org/packages/2.9/bioc/html/limma.html) was used to process datasets GSE189993 and GSE141024 desperately. Background correction and normalization were applied to the expression matrices, followed by gene annotation and extraction of protein-coding genes, which yielded sample-specific gene expression matrices for both GSE189993 and GSE141024. The two datasets were then merged and their batch effects were removed using the R package *sva* (Version 3.50.0, http://www.bioconductor.org/packages/2.9/bioc/html/limma.html). The combined dataset, composed of 25 MMD samples and 15 control samples, was regarded as training set for the subsequent analysis.

The 20 PTM gene sets were merged and deduplicated to serve as a PTM set for further analysis (Tables 1, 2).

**TABLE 1:** Introduction of dataset.

Dataset	MMD	Control	GPL	Set
GSE189993	21	11	GPL16699	Training set
GSE141024	4	4	GPL16699	Training set
Independently generated dataset	10	3	​	Test set

**TABLE 2:** Post-translational modification gene sets.

Post-translational modification gene sets	Gene set source
Acetylation	PMID40010763
Succinylation	PMID40010763
Malonylation	PMID40010763
Crotonylation	PMID40010763
B-hydroxybutyrylation	PMID40010763
Lactylation	PMID37242427
Palmitoylation	PMID40010763
Myristoylation	PMID40010763
Ubiquitination	REACTOME_PROTEIN_UBIQUITINATION
Sumoylation	REACTOME_SUMOYLATION
Neddylation	REACTOME_NEDDYLATION
ISGylation	PMID40010763
ATG8ylation	PMID40010763
FAT10ylation	PMID40010763
UFMylation	PMID40010763
Methylation	PMID40010763
Glycosylation	PMID40010763
Biotinylation	PMID40010763
S-nitrosylation	PMID40010763
Phosphorylation	PMID40010763
Deubiquitination	REACTOME_DEUBIQUITINATION

The 21 gene sets correspond to 20 types of PTMs, since PMID40010763 regards ubiquitination and deubiquitination as the same category. The number of PTM, gene sets listed in the table above is consistent with the number of gene sets in the literature. Additionally, to ensure the feasibility of subsequent analyses, the Lactylation, Ubiquitination, Sumoylation, Neddylation, and Deubiquitination gene sets were expanded.

### 2.3 Differential expression analysis

Differential expressions were analyzed in the training sets. The gene expressions were compared between MMD and controls using the classical Bayesian and linear regression methods provided by the R package *limma* (version 3.58.1, http://www.bioconductor.org/packages/2.9/bioc/html/limma.html). Differential expressed genes (DEGs) were identified based on both fold change and statistical significance, with the threshold criteria set as |log2FC| >0.263, p < 0.05. Genes which meet these criteria were considered differentially expressed between MMD and controls and were designated as DEGs.

### 2.4 Identification of disease-associated genes using WGCNA

Weighted Gene Co-expression Network Analysis (WGCNA) is a method used to analyze gene expression patterns across multiple samples. It clusters genes with similar patterns and analyzes the association between modules and specific traits or phenotypes.

For the training set, the WGCNA analysis was performed using R package *WGCNA* (Version 1.72–5, https://cran.r-project.org/web/packages/WGCNA/) with the sample sets as phenotypic trait and the full gene expression matrix as input, to examine the MMD-associated genes.

First, sample clustering was processed, followed by network topology analysis based on the *pickSoftThreshold* function. The soft thresholding power and scale-free fit indices (ranging from 1 to 20) were calculated to achieve scale-free topology. And the proper soft threshold was selected based on R2, a scale-free fit index, >0.85. Then, the adjacency matrix was computed according to the selected soft threshold to construct the Topological Overlap Matrix (TOM). Values in the TOM represent the similarity of co-expression between two genes. Next, hierarchical clustering was performed using the flashClust function, and the gene clusters were identified based on the dynamic tree cut algorithm, with the parameter minModuleSize set to 100 (minimum number of genes per module). Each module was randomly assigned a color, and the module eigengenes were calculated via principal component analysis. Finally, the modules which presented significant positive association with MMD were identified as key modules, by calculating the associations between module eigengenes and phenotypic trait. Genes in these key modules were designated as WGCNA, which were considered MMD-related genes by WGCNA.

### 2.5 Identification of DEGs-WGCNA-PTM related genes

The R package *ggvenn* (version 0.1.10, https://mirrors.pku.edu.cn/CRAN/web/packages/ggvenn/index.html) was used to identify the intersection among the DEGs above, WGCNA, and PTM genes. The intersection genes were defined as PTM-related DEGs.

### 2.6 Functional enrichment analysis

To explore the potential biological pathways and functions of PTM-related DEGs, the Gene Ontology (GO) analysis was performed to identify the enrichment of biological process (BP), cellular component (CC), and molecular function (MF) terms in the PTM-related DEGs above, using the R package *clusterProfiler* (Version 4.10.1, https://bioconductor.org/packages/release/bioc/html/clusterProfiler.html). The Kyoto Encyclopedia of Genes and Genomes (KEGG) enrichment analysis was also performed.

### 2.7 Protein-protein interaction (PPI) analysis

The STRING database (Version 12.0, http://www.string-db.org/) was used to construct a PPI network for PTM-related DEGs. The analysis was performed with the species set to *Homo sapiens* and the minimum interaction score threshold set at 0.15. The results were exported and visualized as a PPI network using Cytoscape software (Version 3.9.1).

### 2.8 Machine learning

The features of PTM-related DEGs were selected based on training sets, using Random Forest and Elastic Net. The random forest model was constructed using the randomForest package (Version 4.7–1.2, https://cran.r-project.org/web/packages/randomForest/index.html). Feature importance scores were visualized with the ggplot2 package (Version 3.5.2, https://cran.r-project.org/web/packages/ggplot2/index.html). The R package *glmnet* (Version 4.1–8, https://glmnet.stanford.edu) was used for resilient network regression, with family = “biomial”, alpha set at 0.1, and 10-fold cross-validation for feature selection. The results were visualized using “plot” function.

Genes selected by both machine learning algorithms were intersected, with the intersected genes identified as candidate feature genes.

### 2.9 Feature gene expression and ROC validation

Using the independently generated dataset as test set, expression data of candidate feature genes were extracted from both the training and test sets. The Wilcoxon rank-sum test was applied to assess the significance of differential expression between the MMD and control groups, with a significant threshold set at p < 0.05. Genes representing consistent expression trends in both training and test sets were selected to generate Receiver Operating Characteristic (ROC) curves, by using *pROC* package (Version 1.18.5, https://cran.r-project.org/web/packages/pROC/index.html). The Area Under the Curve (AUC) was calculated to quantify the predictive performance of each gene. Genes exhibiting consistent expression trends, with an AUC value greater than 0.7, in both training and test sets were defined as final feature genes.

### 2.10 SHAP model interpretation

An xgboost model was constructed based on the expression levels of feature genes in the training sets. The model was interpreted by SHAP (SHapley Additive exPlanations) method, using *shapviz* package (Version 0.10.2, https://cran.r-project.org/web/packages/shapviz/index.html), encompassing both global and local interpretation approaches. The SHAP values were calculated by “shapviz” function. The mean contribution of each feature gene to the model was illustrated by a SHAP summary plots, which was plotted by “sv_importance” function, to characterize the overall behavior of the model. A beeswarm plot of SHAP values was generated using the kind = “beeswarm” parameter within the sv_importance function. For local interpretation, force plots and prediction decomposition waterfall plots were employed to explain individual predictions for specific cases.

### 2.11 Correlation between immune infiltration and feature genes

To investigate the immune infiltration profiles of MMD in MMD and control groups, the ssGSEA algorithm implemented in the gsva function from the *GSVA* package (Version 2.2.0, https://www.bioconductor.org/packages/release/bioc/html/GSVA.html) was used to evaluate immune activity of each sample in the training sets. Wilcoxon test was used to identify immune cells exhibiting significant differences between the two groups (p < 0.05). The *psych* package (Version 2.4.1, https://cran.r-project.org/package=psych) was used to analyze the correlations both among immune cells and between the identified feature genes and immune cells with Spearman correlation analysis. The results of correlations were visualized as a heatmap using the *pheatmap* package (Version 1.0.12, https://cran.r-project.org/web/packages/pheatmap/index.html).

### 2.12 Analysis of feature genes and MMD-associated genes

MMD-associated genes were retrieved from the GeneCards database (https://www.genecards.org/) using the keyword “Moyamoya disease”. Expression data of MMD-associated genes were extracted from the training sets, and the Wilcoxon rank-sum test was applied to compare the difference of MMD-associated genes between MMD and control groups. Spearman correlation analysis was used to assess the correlation between the identified feature genes and differentially expressed MMD-associated genes. The results were exhibited as heatmap and scatter plots.

### 2.13 Single gene enrichment analysis

The MMD samples were divided into high- and low-expression groups according to the median expression level of feature genes. The *limma* package was applied to analyze the differential expression between the two groups. All genes were ranked in a descending order based on their log_2_ fold change (LogFC). Next, the KEGG pathway enrichment analysis was performed to identify the activated or inhibited signal pathway in MMD, with the *clusterProfiler* package and the corresponding subsets in MSigDb database “c2.cp.kegg.v2023.1.Hs.symbols.gmt” as the background reference. Significantly enriched pathways were identified using an adjusted p-value threshold of <0.05, and the enrichment results were exhibited accordingly.

### 2.14 Analysis of transcriptional regulation of feature genes

*RcisTarget* (Version 1.23.1, https://bioconductor.org/packages/release/bioc/html/RcisTarget.html) is an R package designed for constructing gene regulatory network and analyzing transcription factors (TFs), which identifies potential TF regulatory networks from a set of genes. Using “hg19-500bp-upstream-7species.mc9nr.genes_vs._motifs.rankings.feather” as the motif Ranking dataset and “motifAnnotations_hgnc” as the Motifs annotation dataset, the TFs of feature genes were predicted by applying cisTarget function. High-confidence TFs with a normalized enrichment score (NES) > 4 were selected to construct the regulatory network.

### 2.15 Construction of diagnostic Gene-microRNA network

The miRDB database (https://mirdb.org/) was applied to predict miRNAs which interact with the identified feature genes to explore the regulatory mechanisms in MMD. When a large number of miRNAs were to be predicted, the top 10 miRNA ranked by “Target Score” were selected to construct mRNA-miRNA network, visualized by Cytoscape.

### 2.16 Analysis of GeneMANIA

A PPI analysis was conducted among the identified feature genes and 20 interacting genes using the GeneMANIA database (http://genemania.org/), to predict the associations of colocalization, shared protein domains, co-expression, genetic interactions, and pathway involvement.

### 2.17 Small molecule drug prediction

The information of interactions between feature genes and small molecule compounds was retrieved from the DGIdb database (Drug-Gene Interaction Database, http://www.dgidb.org). When a plenty of drugs were identified, the most promising small molecule drug was selected based on their interaction score.

### 2.18 Molecular docking

The molecule structure data of all drug active ingredients were retrieved from PubChem database (https://pubchem.ncbi.nlm.nih.gov) and stored in SDF format. The protein structures corresponding to the feature genes were obtained from the RCSB Protein Data Bank (RCSB PDB, http://www.rcsb.org/) and stored in PDB format as protein receptors. The AutoDock Vina software was applied for docking between selected small molecule drugs and feature gene-encoded protein, with the binding energy recorded (a binding energy ≤−5.0 kcal/mol is generally considered indicative of strong binding). The receptor-ligand docking complexes with the strongest binding affinity for each feature gene were filtrated with the results visualized by using PyMOL and Discovery Studio.

### 2.19 Molecular dynamics simulation

Molecular dynamics (MD) simulation was performed to identify the interactions between feature genes and small molecule drug, in which the molecular docking with lowest binding energy was filtrated for further validation. According to the docking results, a 100 ns MD simulation was performed using GROMACS 2024.2 software to further validate the stability and reliability of the docking results. The AMNER gaff force field was generated by the *sobtop* program (Tian Lu, Sobtop, Version 1.0 dev5, http://sobereva.com/soft/Sobtop), while the parameter and topological files of protein and small molecule ligand were derived from the built-in “AMBER14SB” force field in GROMACS 2024.2. Periodic boundary conditions were settled and optimized to simulate the size of limit box and fill the box with water molecules. To maintain electricity neutrality, a few water molecules were replaced with Na^+^ and Cl^−^ ions at a concentration of 0.15 mol/L. The steepest descent algorithm was conducted to achieve energy minimization in the whole system. System pre-equilibration was performed in two phases: first, stabilize the system temperature under NVT system at 300 K and 100 ps; second, stabilize the system pressure under NPT system at 1 bar and 100 ps? The results were visualized using the *DuIvyTools* package (https://duivytools.readthedocs.io/en/v0.6.0/DIT.html).

### 2.20 *In vitro* experimental verification

For validation, peripheral venous blood samples were obtained from three Moyamoya disease patients and three healthy controls. The study protocol was reviewed and approved by the Ethics Committee of Peking Union Medical College Hospital (Beijing, China; I-24PJ2435), and written informed consent was secured from all donors.

The levels of FBXW7 in serum samples were measured using the Human F-Box and WD Repeat Domain Containing 7 (FBXW7) ELISA Kit (abx387334; Abbexa, Texas, United States) following the manufacturer’s protocol.

Small interfering RNAs (siRNAs) targeting FBXW7 and the negative control siRNA (si-NC) were synthesized by Born Biotechnology Co., Ltd. (Nanjing, China). The sequences were as follows:

- si-NC: sense, 5′-UUC​UCC​GAA​CGU​GUC​ACG​UTT-3′; antisense, 5′-ACG​UGA​CAC​GUU​CGG​AGA​ATT-3′;
- si-FBXW7#1: sense, 5′-GGU​CAG​CAG​UCA​CAG​GCA​AAU-3′; antisense, 5′-UUG​CCU​GUG​ACU​GCU​GAC​CAA-3′;
- si-FBXW7#2: sense, 5′-CAA​GUG​GAA​UGG​AAC​UCA​AAG-3′; antisense, 5′-UUG​AGU​UCC​AUU​CCA​CUU​GUU-3′;
- si-FBXW7#3: sense, 5′-CC AUG​UUC​AGC​AAC​ACC​AAC​A-3′; antisense, 5′-UUG​GUG​UUG​CUG​AAC​AUG​GUA-3′.

For siRNA transfection, human brain vascular smooth muscle cells (HBVSMCs) were seeded into 6-well plates at a density of approximately 2 × 10^5^ cells per well and cultured for 24 h in antibiotic-free medium until reaching 70%–90% confluence. Prior to transfection, cells were serum-starved for 1–2 h and then maintained in serum-free Opti-MEM medium (total volume = 2 mL per well).

The transfection complex was prepared as follows: 5 µL Lipofectamine 3000 (Thermo Fisher Scientific) was gently mixed with 250 µL serum-free medium and incubated for 5 min at room temperature. Separately, 10 µL siRNA (final concentration = 20 µM) was diluted in 250 µL serum-free medium and also incubated for 5 min. The two solutions were then combined and incubated for 20 min at room temperature to form the siRNA–Lipofectamine 3000 complex.

Before transfection, the culture medium was replaced with fresh antibiotic-free medium. Then, 50 µL of the siRNA–Lipofectamine complex was added dropwise to each well and gently mixed. Cells were incubated under standard conditions (37 °C, 5% CO_2_) for 6 h, followed by medium replacement with complete growth medium. Transfection efficiency was verified by qRT-PCR, and si-FBXW7#3 was selected for subsequent experiments (Supplementary Figure S12).

### 2.21 Cell culture conditions

Human brain vascular smooth muscle cells (HBVSMCs) were cultured in Smooth Muscle Cell Medium (SMCM; ScienCell, United States) supplemented with 2% fetal bovine serum (FBS), 1% penicillin–streptomycin (P/S), and 1% Smooth Muscle Cell Growth Supplement (SMCGS). When the cultures reached approximately 90% confluence, the medium was removed and the cells were washed twice with phosphate-buffered saline (PBS, 2 mL each). Cells were then dissociated using 2 mL of 0.25% trypsin containing 0.02% EDTA. The digestion process was carefully monitored under a microscope and terminated after about 30 s, once the cells became rounded, by adding an equal volume of complete culture medium. The cells were gently pipetted to achieve a single-cell suspension, collected, and centrifuged at 800 rpm for 5 min at 4 °C. After discarding the supernatant, the pellet was resuspended in fresh complete medium and seeded into new culture flasks. The culture medium was replaced every 2 days to maintain optimal cell growth.

### 2.22 Scratch assay

The migratory ability of HBVSMCs was assessed using a wound healing assay. Briefly, straight reference lines were drawn on the back of a sterile 6-well plate using a UV-sterilized marker at intervals of 0.5–1 cm, crossing the center of each well. Cells in the logarithmic growth phase were trypsinized, resuspended in complete medium, and seeded at a density of 6 × 10^5^ cells/mL (2 mL per well) to ensure confluence after 24 h. After incubation at 37 °C with 5% CO_2_ for 24 h, a uniform scratch was made perpendicular to the reference lines using a sterile 200 μL pipette tip. The wells were gently washed three times with PBS to remove detached cells, and serum-free medium was added. The plates were then incubated under standard conditions (37 °C, 5% CO_2_). Images were captured at 0 h and subsequent time points under a light microscope at ×100 magnification, ensuring consistent background and scratch orientation. The wound width was analyzed using ImageJ software to evaluate cell migration.

### 2.23 EdU assay

HBVSMCs were seeded into 96-well plates at a density of 1 × 10^4^ cells per well and cultured overnight. After the indicated treatments, cells were incubated with 10 μM EdU (prepared by diluting the stock solution in culture medium) for 2 h at 37 °C. The cells were then fixed with 4% paraformaldehyde for 15 min at room temperature, washed three times with PBS, and permeabilized with 0.3% Triton X-100 for 15 min.

After washing, the Click reaction mixture was added and incubated for 30 min in the dark, following the manufacturer’s instructions. The nuclei were stained with Hoechst 33342 (1:1000 in PBS) for 10 min, and the cells were washed again with PBS. The stained cells were observed and photographed under a fluorescence microscope to evaluate EdU-positive cells.

### 2.24 Statistical analysis

All data were analyzed and plotted using GraphPad Prism 9 (version 9.4.0), and figures were arranged with Adobe Illustrator 2022. Results are presented as mean ± SD. Statistical differences between groups were determined by one-way ANOVA followed by Tukey’s *post hoc* test. A *P* value <0.05 was considered statistically significant.

## 3 Results

### 3.1 Data

The GSE189993 dataset comprises 21 MMD samples and 11 control samples, while GSE141024 dataset comprises 5 MMD samples and 4 control samples. The independently generated datasets included 10 MMD STA tissue samples and 3 control samples. 1220 PTM-related genes were obtained by merging and deduplicating 20 PTM gene sets.

### 3.2 Identification of DEGs

**FIGURE 1:**
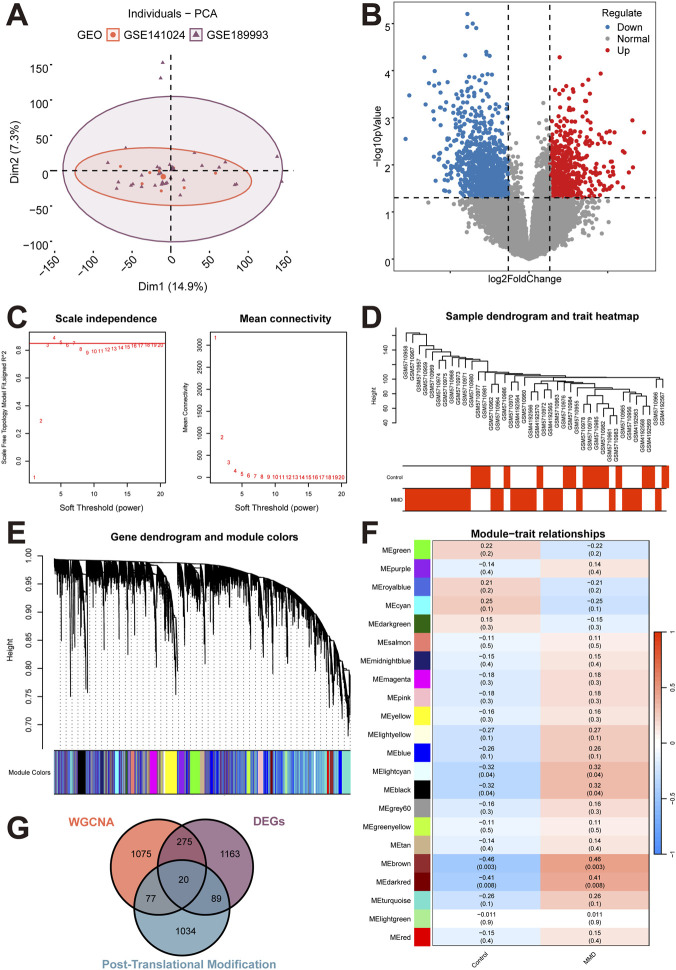
Data Preprocessing and Differential Analysis. **(A)** PCA distribution of samples after batch correction. The boxplots of samples before and after batch correction are displayed in Supplementary Figures S1A, S1BA. **(B)** Differential Expressed Genes volcano plot. Red dots indicate upregulated genes, blue dots indicate downregulated genes, the horizontal black dashed line corresponds to the adjusted p-value threshold of 0.05, and the vertical black dashed lines correspond to |log_2_FC| = 0.263. The differential expressed gene heatmap is displayed in Supplementary Figure S1C. **(C)** Analysis of scale-free index with soft threshold power (β) ranging from 1 to 20. In both panels, the horizontal axis represents the power value. The vertical axis in the left panel represents the Scale Free Topology Model Fit (signed R^2^), in which the higher R^2^ values indicate closer approximation of the network to a scale-free distribution. The vertical axis in the right panel denotes the mean connectivity of genes among all the corresponding modules. **(D)** Phenotypic sample clustering dendrogram. The upper portion represents the sample clustering dendrogram, while the lower portion represents the phenotypic information of corresponding samples. **(E)** Hierarchical clustering dendrogram. The upper portion displays hierarchical clustering dendrogram of genes, while the upper portion represents the gene modules, i.e., the network modules. These two portions are aligned, exhibiting that genes clustering together (proximity) are assigned to the same module. Each color represents a distinct module, while the gray module represents not assigned to any specific module. **(F)** Heatmap of correlation between modules and phenotypical traits. The horizontal axis represents the traits, while the vertical axis represents different gene modules. The main body of the heatmap displays the correlation heatmap, with blue indicating negative correlations and red indicating positive correlations. In each cell, the numerical values denote correlation coefficient, that the higher absolute values indicate stronger associations. The value in parentheses represents the significance, p-values, that the smaller values indicate stronger statistical evidence for the correlation. **(G)** Intersecting genes.

The GSE189993 and GSE141024 datasets were merged. Principal component analysis (PCA) and boxplots were generated to visualize the data distribution before and after batch correction. The PCA distribution of samples after batch correction is shown in Figure 1A. The merged dataset comprised 25 MMD samples and 15 control samples, and was used as training set for subsequent analyses.

Differential expression analysis between MMD and control groups was performed on the training set, with a threshold of |log_2_FC| > 0.263 and p < 0.05. A total of 1,547 DEGs were identified, including 588 upregulated and 959 downregulated genes. The corresponding volcano plot is shown in Figure 1B.

### 3.3 Identification of disease-associated genes via WGCNA analysis

WGCNA analysis was performed on 17596 genes from 40 samples. A soft threshold was selected as 4 based on a scale-free fit index R^2^ > 0.85, with R^2^ achieved 0.8903936, as shown in Figure 1C. The inter-sample similarity and phenotypic differences were visualized by integrating sample clusters and phenotypic information, as shown in Figure 1D. Genes were clustered to generate a hierarchical clustering dendrogram, with the parameter minModuleSize set at 100 (minimum of 100 genes per module), as shown in Figure 1E. A total of 22 gene modules were obtained. Based on module-MMD trait correlations and p value, the brown module was selected due to its significant positive correlation with MMD trait, with cor = 0.46 and p < 0.05 (Figure 1F). 1447 genes were comprised in the brown module, which was identified as MMD-associated genes by WGCNA analysis.

### 3.4 Identification of DEGs-WGCNA-PTM-related genes

A Venn analysis was performed to identify DEGs, WGCNA, and PTM-related genes by using R package *ggvenn*. 20 intersected genes were obtained, designated as PTM-related DEGs, as shown in Figure 1G.

### 3.5 Enrichment analysis

**FIGURE 2:**
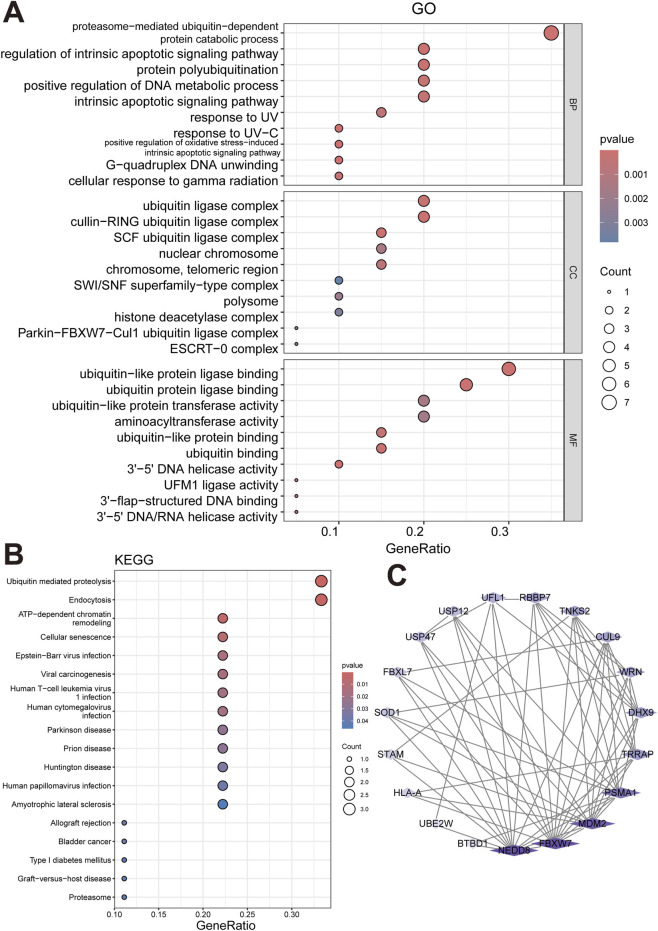
Functional enrichment and protein–protein interaction (PPI) analysis of PTM-related differentially expressed genes in Moyamoya disease. **(A)** Gene Ontology (GO) enrichment analysis, including Biological Process (BP), Cellular Component (CC), and Molecular Function (MF) categories. **(B)** Kyoto Encyclopedia of Genes and Genomes (KEGG) pathway enrichment analysis. **(C)** Protein–protein interaction (PPI) network constructed from 20 PTM-related differentially expressed genes using the STRING database.

GO and KEGG analyses were performed on the 20 PTM-related DEGs. 29 pathways were enriched in GO_BP. Several pathways were significantly enriched, including “proteasome-mediated ubiquitin-dependent protein catabolic process”, “G-quadruplex DNA unwinding”, and “positive regulation of oxidative stress-induced intrinsic apoptotic signaling pathway” (Figure 2). For GO_CC, 22 terms were enriched, such as “cullin-RING ubiquitin ligase complex”, “SCF ubiquitin ligase complex”, and “ubiquitin ligase complex”. As for GO_MF, 69 terms were enriched, notably “ubiquitin-like protein ligase binding”, “ubiquitin protein ligase binding”, and “3′-5′ DNA helicase activity”. 18 pathways were enriched in KEGG analysis, under p < 0.05, where pathways including “Ubiquitin-Mediated Proteolysis”, “Endocytosis”, and “ATP-dependent chromatin remodeling” were significantly enriched.

### 3.6 PPI network construction

PPI network was constructed using 20 PTM-related DEGs, as shown in Figure 2C.

### 3.7 Candidate biomarkers selection via machine learning

**FIGURE 3:**
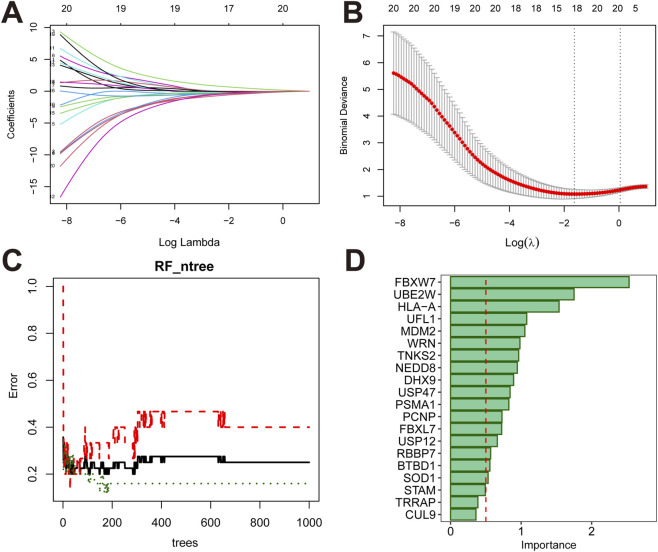
Determination of feature number in Elastic Net regression and RF. **(A)** The coefficient path plot. **(B)** Cross-validation curve of the Elastic Net regression. The X-axis represents the logarithm of the penalty parameter, log(λ). And the Y-axis indicates the binomial deviance. Lower deviance values correspond to better model fit. The numbers at the top indicate the number of remaining variables at different λ values. The two dashed lines represent two specific lambda (λ) values. The left dashed line indicates λ min, which minimizes the deviance and yields the optimal model fit. The right dashed line represents λ-1SE, indicating the value within one standard error of the minimum λ on the right side. **(C)** The horizontal axis represents the number of ntree, and the vertical axis indicates the error rate. **(D)** The horizontal axis indicates the importance score of the intersected genes, with a red vertical line marking the threshold of 0.5. Genes with importance score greater than 0.5 were identified as feature genes selected by the RF model.

Based on the training set, 18 genes were selected using the Elastic Net regression, as shown in Figures 3A,B. The RF algorithm identified 17 genes, as shown in Figures 3C,D.

The intersection of genes identified by both methods yielded 15 candidate biomarkers: FBXW7, UBE2W, HLA-A, UFL1, MDM2, WRN, TNKS2, DHX9, USP47, PSMA1, PCNP, FBXL7, USP12, BTBD1, SOD1.

Details of the candidate biomarker are displayed in Supplementary Table S5.

### 3.8 Validation of expression level and function of feature genes

**FIGURE 4:**
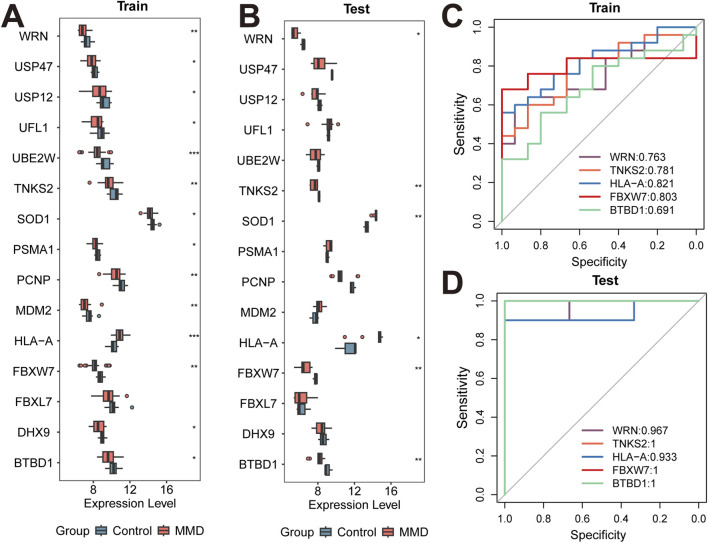
Expression level of candidate feature genes. **(A,B)** Boxplots showing the expression levels of candidate feature genes in the training **(A)** and test **(B)** datasets. (*p* < 0.05, *; *p < 0.01, **p < 0.001) **(C,D)** ROC curves of feature genes from training and test sets. The Area Under the Curve (AUC) values are labeled in the legends, indicating the diagnostic performance of each gene in distinguishing MMD from control samples.

Boxplots were generated based on both training and test sets to exhibit the expression level of candidate feature genes above. Significant differences between groups were identified by Wilcoxon rank-sum test. It was illustrated in Figure 4 that WRN, TNKS2, HLA-A, FBXW7, and BTBD1 exhibited consistent and different expression levels. Among these genes, WRN, TNKS2, FBXW7, and BTBD1 were downregulated in MMD samples while HLA-A was upregulated.

ROC curves were generated based on both training and test sets to illustrate the diagnostic value of genes WRN, TNKS2, HLA-A, FBXW7, and BTBD1 (Figures 4C,D). The results indicate that AUC values of genes WRN, TNKS2, HLA-A, and FBXW7 were greater than 0.7 in both training and test sets, demonstrating their favorable performance in diagnose. These four genes were designed as feature genes for subsequent analyses. It is illustrated in the functional annotations that WRN is associated with sumoylation, TNKS2 with deubiquitination, HLA-A with ubiquitination, and FBXW7 with neddylation.

### 3.9 SHAP model interpretation

**FIGURE 5:**
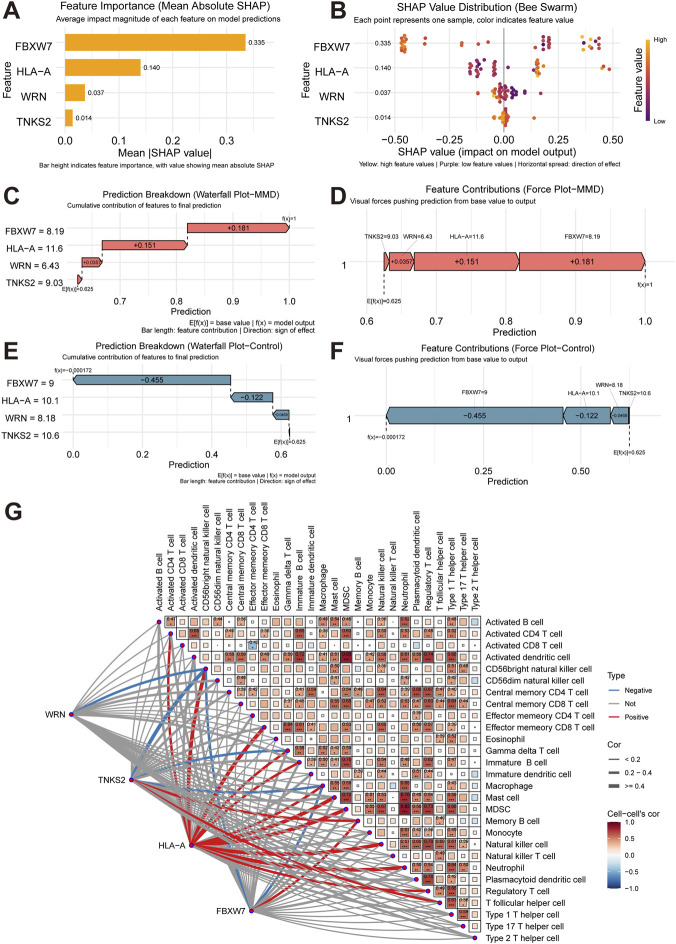
Gene-Immune Cell Correlation. **(A)** Global feature bar plot. **(B)** Global feature beeswarm plot. The Y-axis represents the feature, while the X-axis represents the Shapley value. Colors indicate the feature value (yellow for high and purple for low). Points are jittered along the Y-axis, exhibiting the Shapley value distribution of each feature. Features are ranked based on their importance. **(C,D)** SHAP waterfall plot and SHAP force plot for MMD samples. **(E,F)** SHAP waterfall plot and SHAP force plot for control samples. The numerical values on the arrow indicate the feature values for each specific instance. Features pushing the prediction higher are shown in red, while those pushing it lower in blue. The longer arrow indicates stronger influence on the output. The value on the X-axis quantifies the degree of increase or decrease in prediction influence. **(G)** Heatmap of correlation analyses between feature genes and immune cells. The lower triangular matrix of the heatmap exhibits the correlations between genes and immune cells. Red connecting lines represent significant positive correlations, blue connecting lines represent significant negative correlations, and gray connecting lines denote no significant correlations. The gene-immune cells correlations correspond to the results shown in Supplementary Figure S3. The upper triangular matrix exhibits the correlations between different immune cells. Numerical values in the boxes represent correlation coefficients, and asterisks denote statistical significance. Boxes without numbers indicate no significant correlations between the respective immune cells.

Both global and local approaches of SHAP method were applied to interpretate the model. For global interpretations, the SHAP summary plots were generated to illustrate the mean contribution value of each feature to the model, characterizing the overall function of the model. The result indicated that FBXW7 was the most significant feature, followed by HLA-A, while WRN and TNKS2 ranking lower in importance (shown in Figures 5A,B). As for the local interpretation, individual case analyses were conducted for both MMD and control samples. Visualization of the results illustrated that all these 4 genes positively contributed to the disease prediction (shown in Figures 5C,D). Visualization of control samples analyses results are shown in Figures 5E,F.

### 3.10 Correlations between immune infiltration and diagnostic genes

Immune infiltration analyses were performed. 7 immune cell types exhibited significant differences in infiltration level among MMD and control samples, including CD56bright natural killer cell, immature B cell, macrophage, monocyte, neutrophil, type 1 T helper cell, and type 17 T helper cell (shown in Supplementary Figure S2).

Results in Figure 5G reveal significant cooperative relationships among immune cells. For example, myeloid-derived suppressor cells (MDSC) showed significant positive correlation with activated dendritic cells (r = 0.83). While a significant negative correlation was detected between effector memory CD4 T cells and activated CD8 T cells (r = −0.43).

A significant cooperative relationship was identified between feature genes and immune cells as well (shown in Supplementary Figure S3). FBXW7 was significantly positively correlated with memory B cells, but significantly negatively correlated with CD56bright natural killer cells and macrophages. HLA-A showed significantly positive correlations with activated CD4 T cells, CD56bright natural killer cells, effector memory CD8 T cells, gamma delta T cells, immature B cells, macrophages, mast cells, MDSCs, natural killer cells, neutrophils, and regulatory T cells. TNKS2 was significantly positively correlated with memory B cells and T follicular helper cells, yet negatively correlated with CD56bright natural killer cells, gamma delta T cells, and macrophages. A significant negative correlation was also detected between WRN and CD56bright natural killer cells.

### 3.11 Analyses of correlations between diagnostic genes and MMD

**FIGURE 6:**
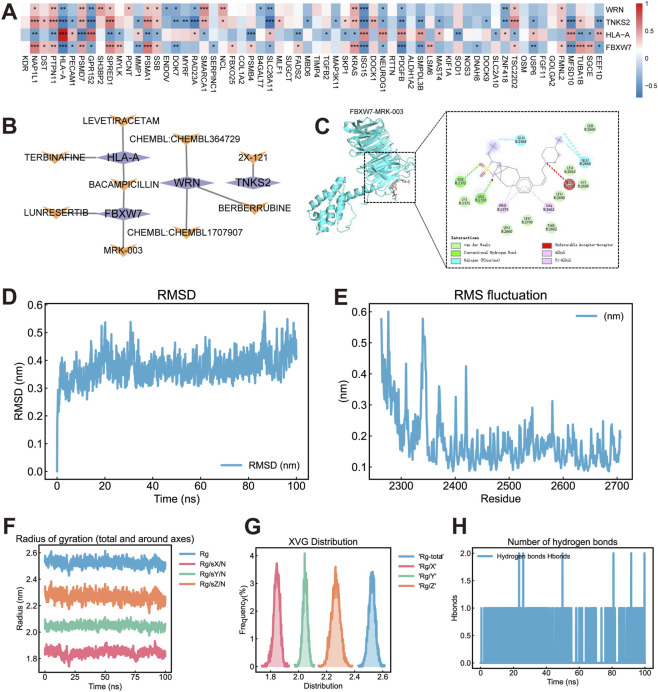
Association Map of MMD-Related Genes. **(A)** Heatmap of correlations between feature genes and MMD-related genes. **(B)** The purple rhombuses represent feature genes, and the orange quadrangles represent the small molecule drugs. **(C)** Visualization of molecule docking result of FBXW7- MRK-003. The left panel of the molecular docking diagram depicts the protein structure in blue ribbon representation, while the small molecule drug is shown in pink. The right panel displays a two-dimensional schematic of the docking interaction results. **(D)** RMSD plot of FBXW7 protein. **(E)** RMSF plot of FBXW7-MRK-003 complex. **(F)** RG plot of FBXW7-MRK-003. **(G)** XVG Distribution for the FBXW7–MRK-003 complex. **(H)** Number of hydrogen bonds plot of FBXW7-MRK-003.

590 MMD-related genes were obtained from GeneCards database. Expression data of 562 of these genes were extracted from the training set, 62 of which exhibited significant differences between MMD and control groups. Sprearman correlation analysis was performed between the 4 feature genes and 62 differentially expressed MMD-related genes. Results in Figure 6A illustrated that the feature genes were significantly related to most of the MMD-related genes. Notably, NAP1L1, PTPN11, HLA-A, PSMD7, GPR152, SPRED1, PSMA1, SSB, SLC26A11, KRAS, ISG15, NEUROG1, PDGFB, and TSC22D2 showed significant correlations with all four feature genes (WRN, TNKS2, HLA-A, FBXW7). The strongest negative correlation was detected between TNKS2 and SLC26A11 (r = −0.71) while the strongest positive correlation was identified between HLA-A and BPR152 (r = 0.67).

### 3.12 Single gene enrichment analysis

Single gene GSEA (Gene Set Enrichment Analysis) was performed. Under an adjusted p-value threshold of <0.05, 10 pathways were enriched for FBXW7, while 12 pathways for HLA-A, 27 pathways for TNKS2, and 41 pathways for WRN. Supplementary Figures S4–S7 exhibits the top 10 pathways ranked by the absolute value of the enrichment score for each individual gene.

### 3.13 Transcriptional regulation of diagnostic genes

Regulatory networks linking feature gene and TF were constructed. The analysis indicated that 17 TF is associated with FBXW7, 7 with HLA-A, 12 with TNKS2, and 4 with WRN (Supplementary Figure S8).

### 3.14 Construction of the diagnostic Gene-microRNA network

A regulatory network between feature genes and miRNA was constructed, where each gene targets 10 miRNA (shown in Supplementary Figure S9).

### 3.15 Analysis of GeneMANIA database

The GeneMANIA database predicted 20 genes interacting with the feature genes, through multiple functional association categories, including PhyMMDal interactions, co-expression, predicted interactions, co-localization, genetic interactions, pathway involvement, and shared protein domains. And a PPI network was constructed (Supplementary Figure S10).

### 3.16 Identification of candidate drugs targeting feature genes

Small molecule drug corresponding to the feature genes were obtained from DGIdb database. Subsequently, a network linking feature genes and small molecule drugs was constructed (Figure 6B).

### 3.17 Molecule docking

Molecule docking was simulated between the feature genes and corresponding small molecule drugs. The results are shown in Table 3. Additionally, the complex with the lowest binding energy for each feature gene was selected and visualized, as shown in Figure 6C; Supplementary Figure S11.

**TABLE 3:** The results of molecule docking.

Gene	pdb_id	Drug	Energy (kcal/mol)
TNKS2	3twr	2X-121	−6.8
FBXW7	5v4b	MRK-003	−8.5
FBXW7	5v4b	LUNRESERTIB	−8.2
FBXW7	5v4b	BACAMPICILLIN	−7.9
HLA-A	7qpd	BACAMPICILLIN	−7.8
HLA-A	7qpd	TERBINAFINE	−6.9
HLA-A	7qpd	LEVETIRACETAM	−5.4
WRN	2fc0	CHEMBL:CHEMBL364729	−4.6
WRN	2fc0	BERBERRUBINE	−6.9
WRN	2fc0	CHEMBL:CHEMBL1707907	−6.8

### 3.18 Molecular dynamics simulation

MD simulations were performed based on the molecular docking results to validate the stability of the interaction between the feature genes and the compound. The MD simulation was processed between FBXW7 and MRK-003 due to the lowest binding energy of this complex. Several key metrics were validated to assess the stability of the complex. The Root Mean Square Deviation (RMSD) represents the distance between atoms of different structures. Protein RMSD indicates conformational changes relative to the initial structure, while the convergence trend of the protein and ligand RMSD assesses whether the simulation has reached stability. Root Mean Square Fluctuation (RMSF) denotes the time-averaged fluctuation of atomic positions, reflecting the flexibility and mobility of the aimino acid throughout the simulation. The Radius of Gyration (Rg) represents the compactness of the protein structure and reflects the changes of tightness of peptide chain during simulation. The hydrogen bond counts indicate the number of hydrogen bonds between proteins and molecules over time. A hydrogen bond was defined when the distance between donor-acceptors was less than 0.35 nm and the hydrogen-donor-acceptor angle <30°. As shown in Figures 6D–H, the results illustrated high flexibility of the protein, stability of FBXW7-MRK-003 complex and the protein peptide throughout the simulation process.

### 3.19 Biological role of FBXW7 in vascular smooth muscle cells

**FIGURE 7:**
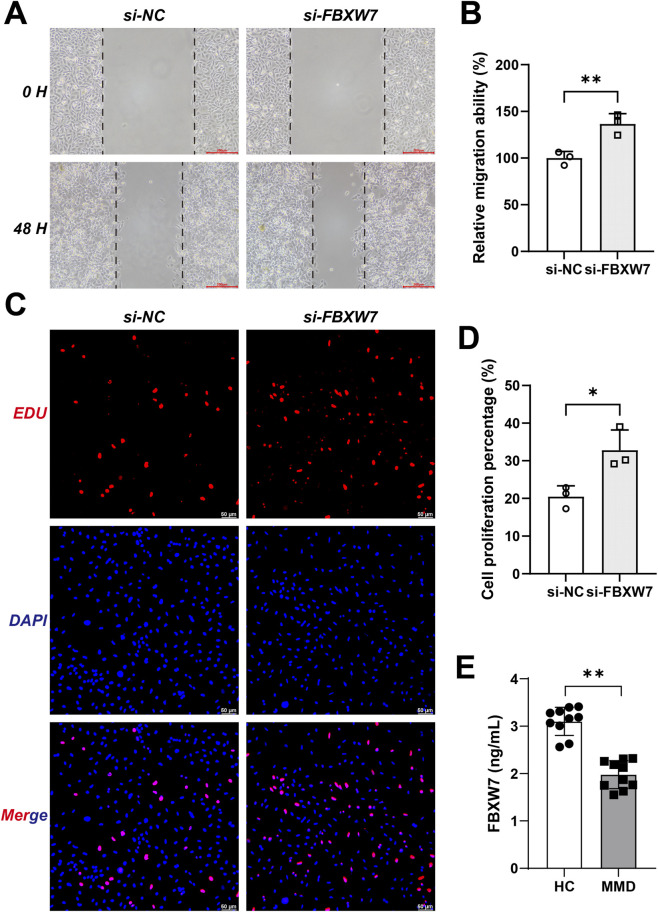
FBXW7 downregulation enhances vascular smooth muscle cell migration and proliferation, and its serum level decreases in MMD. **(A)** Representative images of scratch assays in HBVSMCs transfected with si-NC or si-FBXW7 at 0 h and 48 h. **(B)** Quantification of relative migration ability (%) (n = 3). **(C)** Representative EdU staining showing proliferating cells (red) and nuclei (blue) in the si-NC and si-FBXW7 groups. **(D)** Statistical analysis of EdU-positive cell percentage (%) (n = 3). **(E)** ELISA analysis of serum FBXW7 levels in healthy controls (HC) and MMD patients (n = 10 per group). Data are presented as mean ± SD; *P* < 0.05, *P* < 0.01.

To further investigate the biological role of FBXW7 in vascular smooth muscle cells, we performed wound healing and EdU assays after FBXW7 knockdown. As shown in Figures 7A,B, the migration ability of HBVSMCs was significantly increased in the si-FBXW7 group compared with the si-NC group (P < 0.01). Similarly, EdU staining demonstrated that the percentage of proliferating cells was markedly higher in the si-FBXW7 group than in the control group (P < 0.05; Figures 7C,D). In the expanded validation cohort, ELISA measurements demonstrated that serum FBXW7 levels were markedly reduced in patients with Moyamoya disease compared with healthy controls (P < 0.05; Figure 7E). These results indicate that FBXW7 downregulation promotes vascular smooth muscle cell migration and proliferation, which may contribute to vascular remodeling in MMD.

## 4 Discussion

MMD is a chronic disorder characterized by progressive stenosis and occlusion of the intracranial part of internal carotid artery and compensatory smoke-like collateral vessels. Despite advances in surgical treatment, the underlying mechanisms of MMD remain unclear (He et al., 2025a). In this study, we integrated transcription datasets, combined with network analysis and machine learning approaches to identify the role of PTMs in MMD pathogenesis. Notably, FBXW7 was emerged as a central node linking PTM processes, immune cell infiltration, and vascular phenotypes. Based on the bioinformatic results, a computational pharmacology analysis identified that FBXW7-MRK-003 exhibiting stable binding and favorable conformational properties. This provides an initial translational angle targeting FBXW7-related pathways in MMD.

Prior research has revealed multiple mechanisms related to MMD, including aberration of angiogenesis and immune dysfunction (Zhou et al., 2025; Weinberg et al., 2011). Large vessels of MMD typically exhibit fibrocellular thickening of the tunica intima with excessive proliferation of the vascular smooth muscle cells, along with distal collateral vascular networks in abnormal angioarchitecture (Takekawa et al., 2004). PTMs regulated by RNF213 have been reported to be related to the pathogenesis of MMD. The mutations in RNF213 primarily disrupt the E3 ligase function, leading to a failure in proteasomal degradation of caveolin-1 (Cav-1) by ubiquitination.

Otten et al. (2021) suggested that RNF213 directly catalyzes the ubiquitylation of lipopolysaccharide (LPS), linking it to host immune defense against bacterial infection (Otten et al., 2021). Recent research has revealed that RNF213 facilitates K63-linked ubiquitination and nuclear translocation of FOXO1, thereby promoting regulatory T cell (Treg) differentiation and immune tolerance (*Nat. Commun.*, 2024). Considering the pivotal role of immune imbalance in Moyamoya disease, this mechanism implies that dysregulated RNF213-mediated ubiquitination could impair Treg function and exacerbate vascular inflammation, potentially linking immune modulation to vascular pathology in MMD (Yang et al., 2024). Furthermore, the phosphorylation of Cav-1 is also influenced by RNF213. The knockdown of RNF213 led to a dramatic increase in phosphorylation of Cav-1 and subsequently influenced the endothelial nitric oxide synthase (eNOS) and nitric oxide (NO) bioavailability. Consequently, these dysregulations contributed to the aberrant vascular signaling and impaired endothelial function in MMD (Choi et al., 2025). In addition, recent findings indicate that RNF213 also mediates hypoxia-induced inflammatory cell death, linking metabolic stress to immune activation and tissue injury (Bhardwaj et al., 2025). Given that chronic hypoperfusion and local hypoxia are hallmark features of Moyamoya disease, this mechanism may suggests that RNF213-driven inflammatory signaling under hypoxic conditions may contribute to vascular injury and remodeling in MMD.

Other PTMs have also elucidated significant influence on angiogenesis. A recent study by Zhou et al. demonstrated that SUMOylation of VEGFR2 at lysine 1270 plays critical role in regulating trafficking between Golgi apparatus and plasma membrane, which influences the switch of subsequent activation of VEGF and angiogenic signaling (Zhou et al., 2018). Besides, emerging evidence supports that PTMs serve as a key mediator in immune-vascular interactions, particularly protein glycosylation. A classic example is that sialyl-Lewis X (sLex) glycans on leukocyte P-selectin bind E-selectin and P-selectin on the endothelium, which regulates emigration and rolling adhesion in the inflammatory responses (Rodgers et al., 2000; Hickey et al., 2000). Interestingly, a dynamic glycosylation modification, O-GlcNAcylation (O-GlcNAc), orchestrates the immune-vascular interactions in different ways at different immune stages. The acute increase of O-GlcNAc in vascular smooth muscle cells (VSMCs) was identified as vasculoprotective for preventing the inflammatory cytokine-dependent VSMC dysfunction and neointima formation via suppression of inducible nitric oxide synthase (iNOS) expression (Hilgers et al., 2012). However, chronic upregulation of O-GlcNAc in VSMCs was proved to be related to vascular contractile dysfunction via the RhoA-ROCK pathway and subsequent arterial stiffness and atherosclerosis (Lima et al., 2011). These elegant mechanisms above highlight the critical role of PTMs in modulating vascular physiopathological processes. However, how PTMs regulate the development of MMD has remained unclear.

In our study, FBXW7 was selected as a core gene bridging immune regulation and vascular function. FBXW7, F-box and WD repeat domain containing 7, is a member of the F-box protein family, which functions as a critical substrate recognition component of the SCF E3 ubiquitin ligase, involving the proteasome-mediated degradation of several proteins in oncogenesis (Yeh et al., 2018). Prior studies have revealed the mediator role of FBXW7 in vasculogenesis and cell migration. Wang et al. demonstrated that knockdown or inhibition of FBXW7 impaired angiogenesis and endothelial barrier integrity, which was consistent both in HUVECs and in experimental animal models. Furthermore, they elucidated that FBXW7 targeted KLF2 for ubiquitination and proteasomal degradation and this process was phosphorylation-dependent (Wang et al., 2013). However, under normoxic and hypoxic conditions, FBXW7 exhibited a negative modulation on cell migration and angiogenesis in human microvascular endothelia cell-1 (HMEC-1) (Flügel et al., 2012). A recent study by Chen et al. suggests the role of FBXW7 appears to be more complex (Chen et al., 2025). The study identified FBXW7 as a downstream effector within the STAT4/miR-223-3p/FBXW7 signaling axis in human retinal endothelial cells (hRECs) under hyperglycemic conditions. The suppression of FBXW7 under hyperglycemic conditions led to enhanced endothelial cell proliferation, migration, and tube formation via Notch1/Akt/mTOR pathway, which jointly contributed to the pathological retinal angiogenesis. Recent studies have further highlighted the biological relevance of neddylation in vascular regulation. Zou et al. demonstrated that neddylation exerts diverse and pivotal roles in endothelial metabolism, redox balance, and angiogenic responses, thereby maintaining vascular homeostasis. In line with these findings, our identification of FBXW7 as a neddylation-associated gene in Moyamoya disease suggests that dysregulated neddylation signaling may contribute to FBXW7-mediated vascular dysfunction and remodeling (Zou and Zhang, 2020).

In addition, we also observed consistent upregulation of HLA-A in MMD samples whose expression was correlated with multiple immune cell subsets, including activated CD4^+^ T cells, NK cells, macrophages, and regulatory T cells. HLA-A is a crucial member of the major histocompatibility complex (MHC) class I molecules, which initiates immune responses by presenting intracellular peptides to cytotoxic T cells (Bjorkman et al., 1987). Endothelial and other vascular cells are capable of presenting antigen and HLA-A is responsible for monocyte recruitment in a P-selectin- and FcγR-dependent way (Valenzuela et al., 2013). Beyond that, previous study demonstrated that the antibody activation of exocytosis depended on the cross-linking HLA class I. It illustrated that HLA activates exocytosis by triggering specific intracellular signal transduction cascades and subsequently increases intracellular calcium. The endothelial exocytosis promotes monocyte/macrophage adhesion and amplifies vascular inflammation, leading to vascular inflammation in transplanted organs (Yamakuchi et al., 2007). Our findings, that HLA-A upregulation and its positive correlations with various immune cells, along with the prior work jointly proved the pivotal role of HLA-A in vascular inflammation regulation, which may contribute to the development of MMD.

Our study provides integrative evidence that PTMs constitute a significant mechanism in the pathogenesis of MMD. FBXW7, HLA-A, WRN, and TNKS2 were selected as feature genes, among which FBXW7 and HLA-A were identified as linking nodes among PTMs, vascular dysfunction, and immune dysregulation. FBXW7 downregulation may promote aberrant vascular smooth muscle cell proliferation, while HLA-A upregulation may activate vascular immunity. Furthermore, the complex, FBXW7-MRK-003, presented satisfying stability through computational simulations, which provide innovative insights into pharmacological interventions of MMD.

Limitations include modest cohort size, and the absence of *in vitro* or *in vivo* validations. The underlying mechanisms of FBXW7 involving in the pathogenesis of MMD are warranted for more experiments to validate causality and therapeutic potential. Moreover, the molecular docking and molecular dynamics simulations presented in this study are computational (*in silico*) predictions rather than experimental confirmations. These findings provide preliminary insights into potential interactions, which require further biochemical and pharmacological validation. Taking together, our study highlights role of PTM-related genes in MMD and indicates FBXW7 as a bridge between immune and vascular dysregulation, offering novel evidence for future translational studies in MMD.

## Data Availability

The datasets presented in this study can be found in online repositories. The names of the repository/repositories and accession number(s) can be found in the article/Supplementary Material.
