# Supplementary material for: Post-translational modification gene signatures implicate FBXW7 in immune and vascular dysregulation of Moyamoya disease
Source: Front Genet. 2025 Dec 18;16:1723233. doi: 10.3389/fgene.2025.1723233 (PMC12755860; doi:10.3389/fgene.2025.1723233)
Supplement: Supplementary file 1 [file DataSheet2.pdf]

Supplementary material

Table S1

Time	Group	Area			Average	SD	migration			Relative migration ability (%)			Average	SD
0 h	si-NC	9454758	9295343	9133572	9294557.667	160594.4402	0.36461039	0.331397131	0.381901626	101.48	92.23	106.29	100	7.145886929
	si-FBXW7	9208976	9455201	9344790	9336322.333	123330.7086	0.447447577	0.525739326	0.498921645	124.53	146.32	138.86	136.57	11.07402817
48 h	si-NC	6007455	6214893	5645446	5955931.333	288198.6947								
	si-FBXW7	5088442	4484230	4682472	4751714.667	307999.9114								

Table S2

Group	Number of cells			EdU Positive Proportion			Cell proliferation percentage (%)			Average	SD
si-NC	305	223	324	65	51	56	21.31	22.87	17.28	20.48666667	2.884516135
si-FBXW7	264	259	268	77	101	81	29.17	39	30.22	32.79666667	5.397835986

Table S3 Detailed clinical information for the participants of ELISA cohort.

No.	Age (years)	Sex	Hypertension	Diabetes	Coronary heart disease	Hyperlipidemia	Groups	Duration of symptoms (months)	Suzuki stage	Subtype of MMD
1	26	F	NO	NO	NO	NO	ICH	4	2	Bilateral
2	35	M	NO	NO	NO	NO	ICH	5	3	Bilateral
3	38	F	YES	NO	NO	NO	ICH	11	3	Bilateral
4	52	M	NO	NO	NO	NO	ICH	14	3	Bilateral
5	43	F	NO	NO	NO	NO	ICH	5	3	Bilateral
6	43	F	NO	NO	NO	NO	TIA	3	3	Bilateral
7	31	M	NO	NO	NO	NO	TIA	24	2	Bilateral
8	34	M	NO	NO	NO	NO	TIA	1	3	Bilateral
9	31	M	NO	NO	NO	NO	TIA	12	3	Bilateral
10	32	F	YES	NO	NO	NO	TIA	18	2	Bilateral
11	35	F	NO	NO	NO	NO	HC	-	-	-
12	29	F	NO	NO	NO	NO	HC	-	-	-

13	41	M	NO	NO	NO	NO	HC	-	-	-
14	45	F	NO	NO	NO	NO	HC	-	-	-
15	39	F	NO	NO	NO	NO	HC	-	-	-
16	25	M	NO	NO	NO	NO	HC	-	-	-
17	36	M	NO	NO	NO	NO	HC	-	-	-
18	49	M	NO	NO	NO	NO	HC	-	-	-
19	41	M	NO	NO	NO	NO	HC	-	-	-
20	28	F	NO	NO	NO	NO	HC	-	-	-

Abbreviations: MMD, moyamoya disease; F, female; M, male; ICH, intracerebral hemorrhage; TIA, transient ischemic attack; HC, Healthy control.

Table S4：Detailed clinical information for the participants of RNA-seq validation cohort.

No.	Age	Sex	Disease	Hypertension	Diabetes	Smoke	Alcohol	Clinical presentation	Duration (mounth)	Suzuki stge	Subtype of MMD
1	45	M	MMD	N	N	N	N	ICH	9	3	Bilateral
2	32	M	MMD	Y	Y	N	Y	ICH	5	4	Bilateral
3	47	M	MMD	Y	Y	Y	Y	TIA	8	4	Bilateral

4	46	M	MMD	Y	N	N	N	TIA	3	3	Bilateral
5	51	F	MMD	Y	N	N	N	TIA	36	3	Bilateral
6	35	F	MMD	N	N	N	N	ICH	5	4	Bilateral
7	36	F	MMD	N	N	Y	N	TIA	0.5	4	Bilateral
8	52	M	MMD	Y	N	N	Y	TIA	3	4	Bilateral
9	52	M	MMD	N	N	N	N	TIA	6	3	Bilateral
10	31	F	MMD	N	N	N	N	TIA	4	3	Bilateral
11	52	M	IA	Y	N	N	N	ICH	N	N	N
12	51	M	IA	N	N	Y	Y	ICH	N	N	N
13	49	F	IA	N	N	N	N	ICH	N	N	N

Abbreviations: MMD, moyamoya disease; F, female; M, male; IA, intracranial aneurysm; ICH, intracerebral hemorrhage; TIA, transient ischemic attack; N, not reported； Y, reported.

Table S5 Overlapping genes

Elastic Net	RF	Overlapping genes
BTBD1	FBXW7	FBXW7
WRN	UBE2W	UBE2W
HLA-A	HLA-A	HLA-A
PCNP	UFL1	UFL1
STAM	MDM2	MDM2
PSMA1	WRN	WRN
MDM2	TNKS2	TNKS2
CUL9	NEDD8	DHX9
FBXL7	DHX9	USP47
USP47	USP47	PSMA1
DHX9	PSMA1	PCNP
TRRAP	PCNP	FBXL7
UBE2W	FBXL7	USP12
USP12	USP12	BTBD1
FBXW7	RBBP7	SOD1
TNKS2	BTBD1	
SOD1	SOD1	
UFL1		

Figure S1 Data preprocessing and normalization of Moyamoya disease transcriptome datasets.

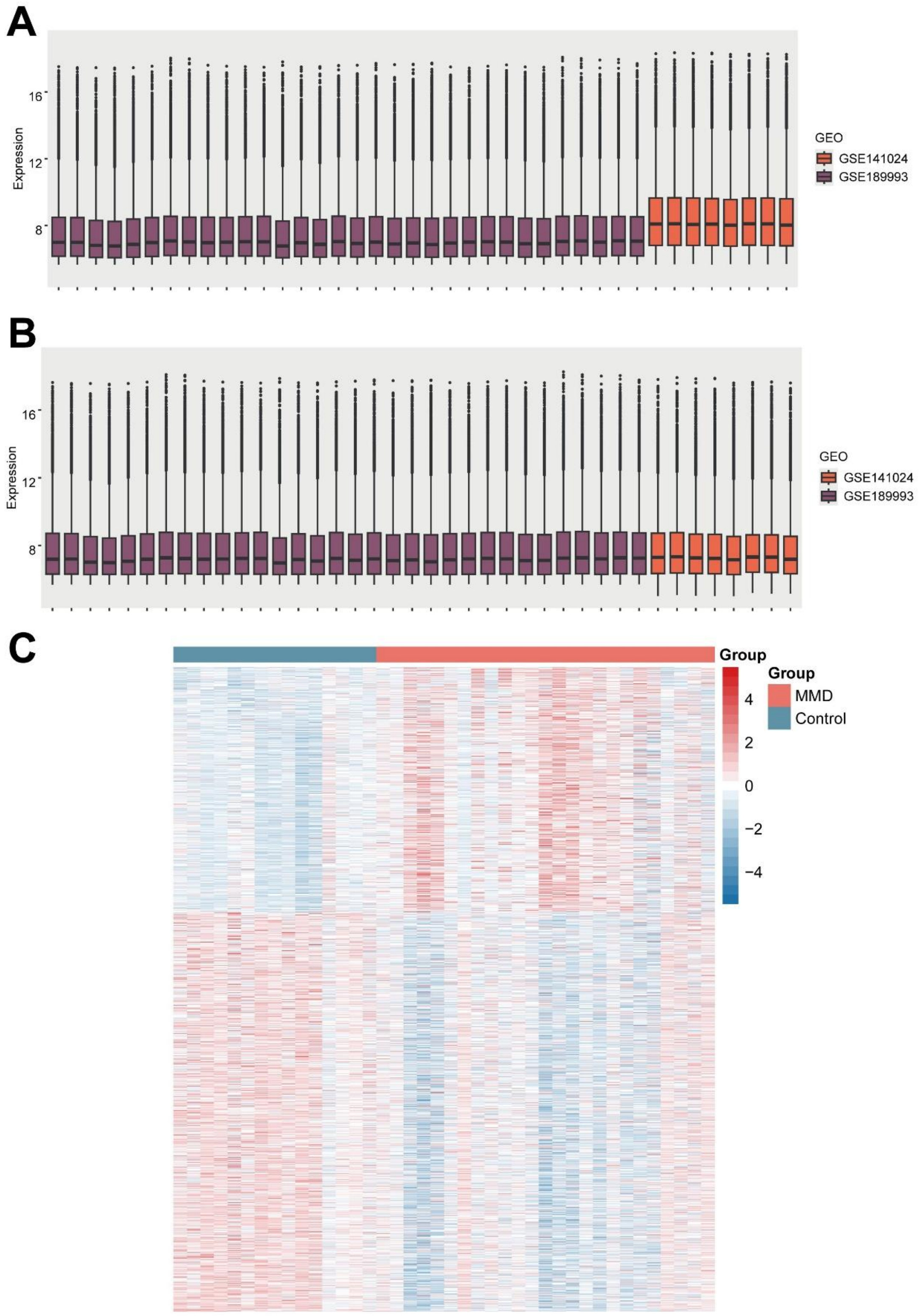

(A) Boxplots showing the expression value distribution of all samples in GSE141024 and GSE189993 datasets before batch correction. Each box represents one sample; the y-axis indicates the normalized expression level.

(B) Boxplots showing the expression value distribution after batch effect removal using the "sva" R package. The consistent median lines across all samples indicate successful normalization and comparability between datasets.

(C) Heatmap of differentially expressed genes (DEGs) between Moyamoya disease (MMD) and control groups after data merging and normalization. Each column represents one sample, and each row represents one gene. Red indicates upregulated expression, and blue indicates downregulated expression. The color bar above the heatmap distinguishes the MMD and control groups.

Figure S2 A: Boxplots for immune infiltration. **B:** Heatmap for correlations between diagnostic genes and immune cells.

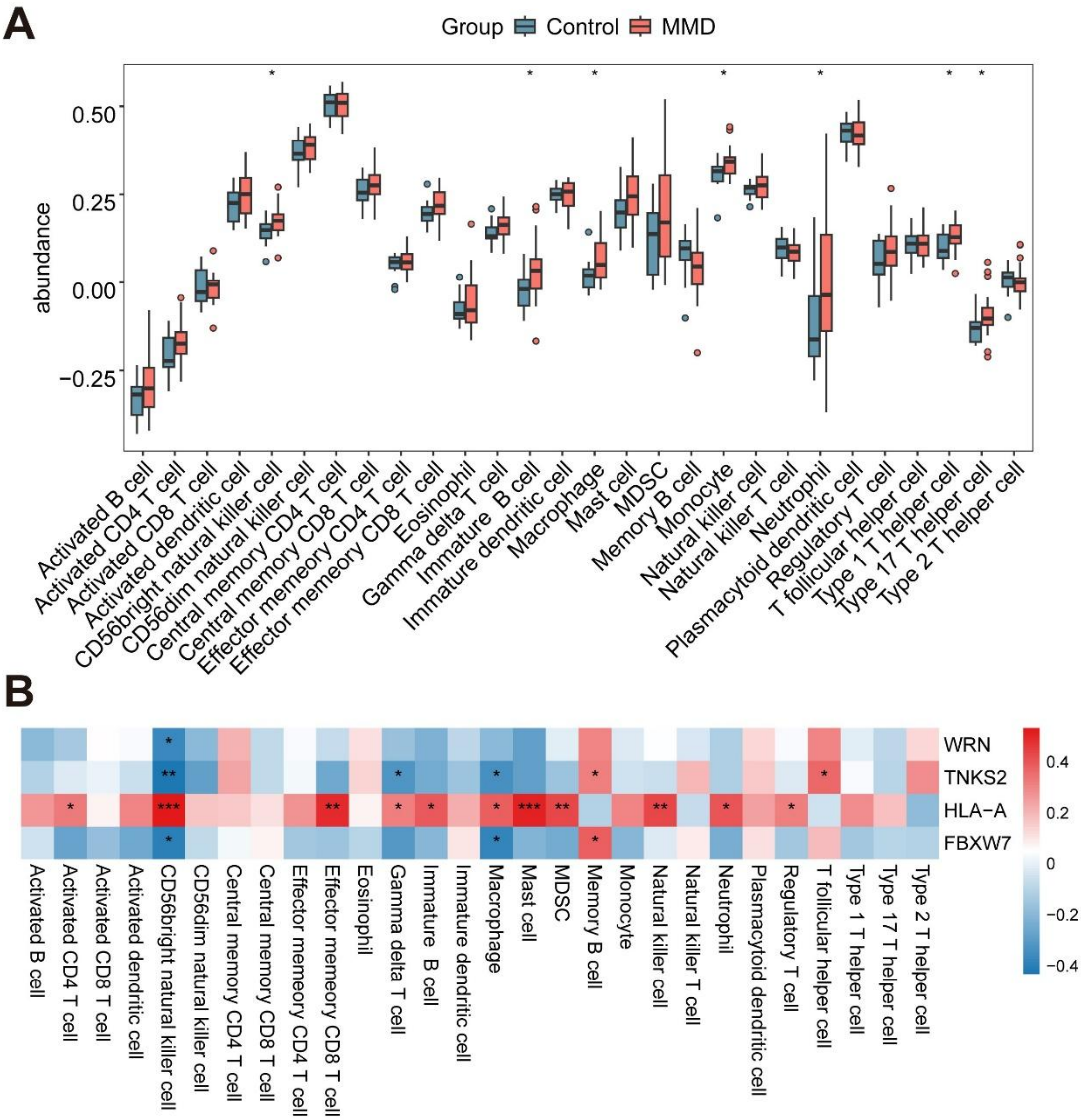

Figure S3 Lollipop plot of gene-immune cell correlation. The vertical axis represents cells while the horizontal axis indicates the strength and direction of correlation between genes and

immune cells. Red lollipops indicate significant positive correlations, blue represents significant negative correlations, and gray indicates non-significant correlations.

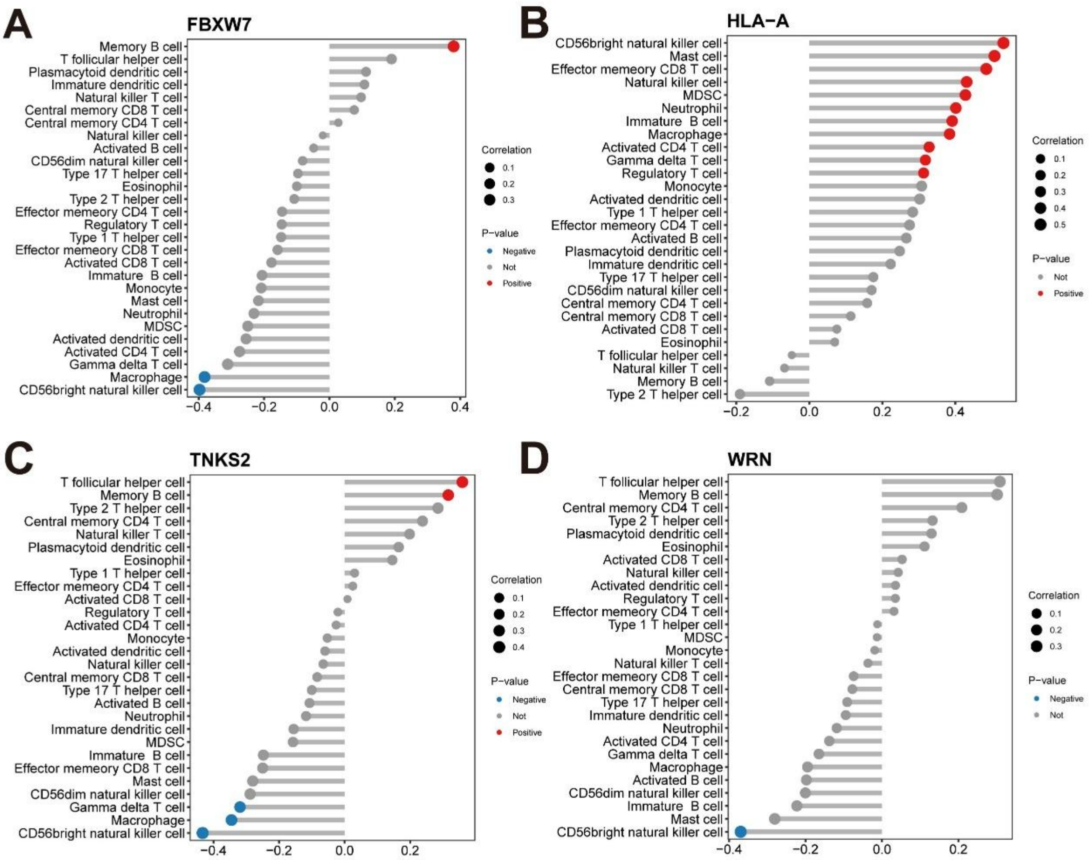

Figure S4. Top 10 pathways ranked by the absolute value of the enrichment score for FBXW7. The top portion is Enrichment Score (ES) Profile Plot, exhibiting the running ES as the analysis progressing through the ranked gene list. The peak value of the curve (the point farthest from the vertical baseline of 0.0) represents the ES for the gene set. In the middle portion of the figure, vertical lines were used to indicate the position in the ranked gene list where members of the gene set are located. Each line marks the existence of a gene from the functional annotation set within the sorted list. The bottom part of the figure is the rank value distribution plot for all genes.

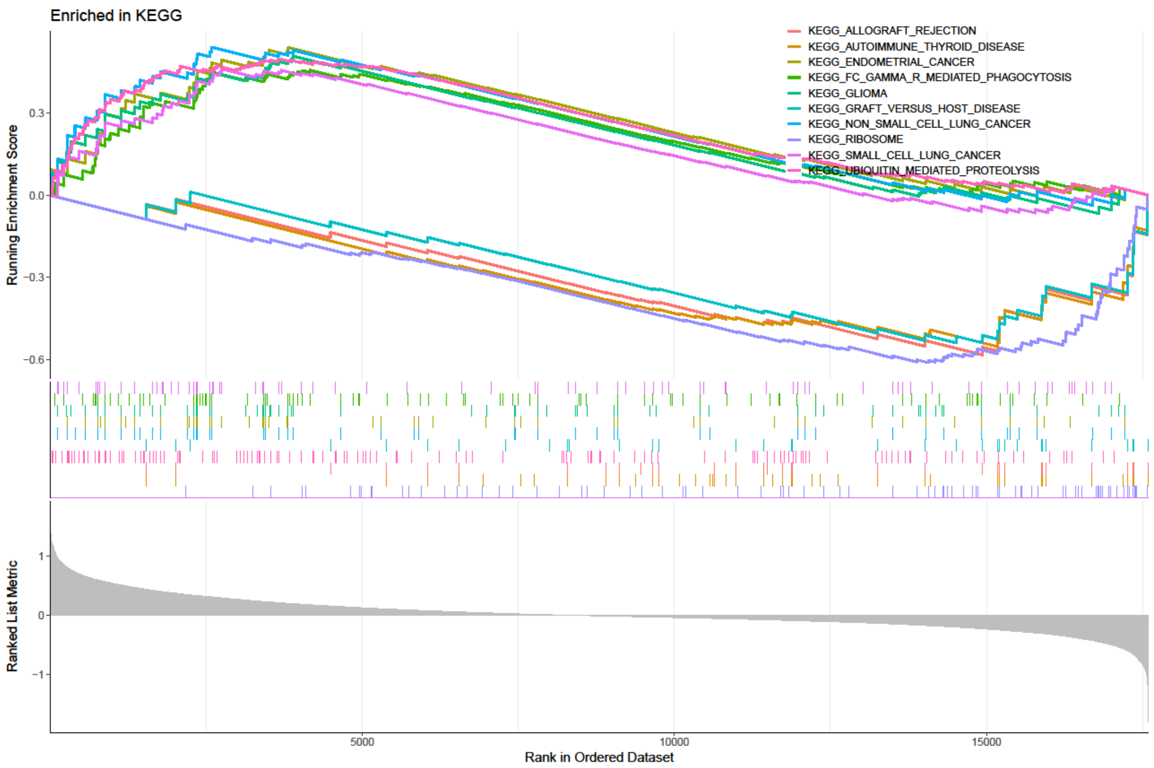

Figure S5. Top 10 pathways ranked by the absolute value of the enrichment score for HLA-A. The illustration of the figure is the same as the illustration of Figure S4.

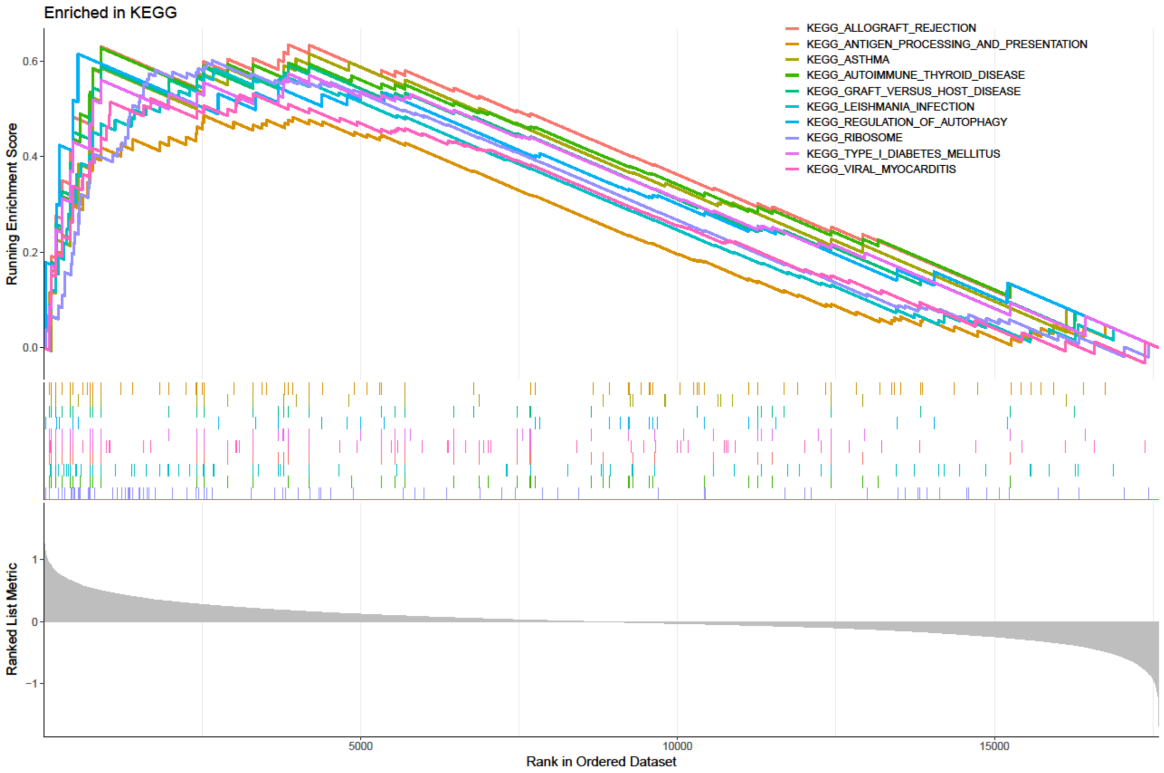

Figure S6. Top 10 pathways ranked by the absolute value of the enrichment score for TNKS2. The illustration of the figure is the same as the illustration of Figure S4.

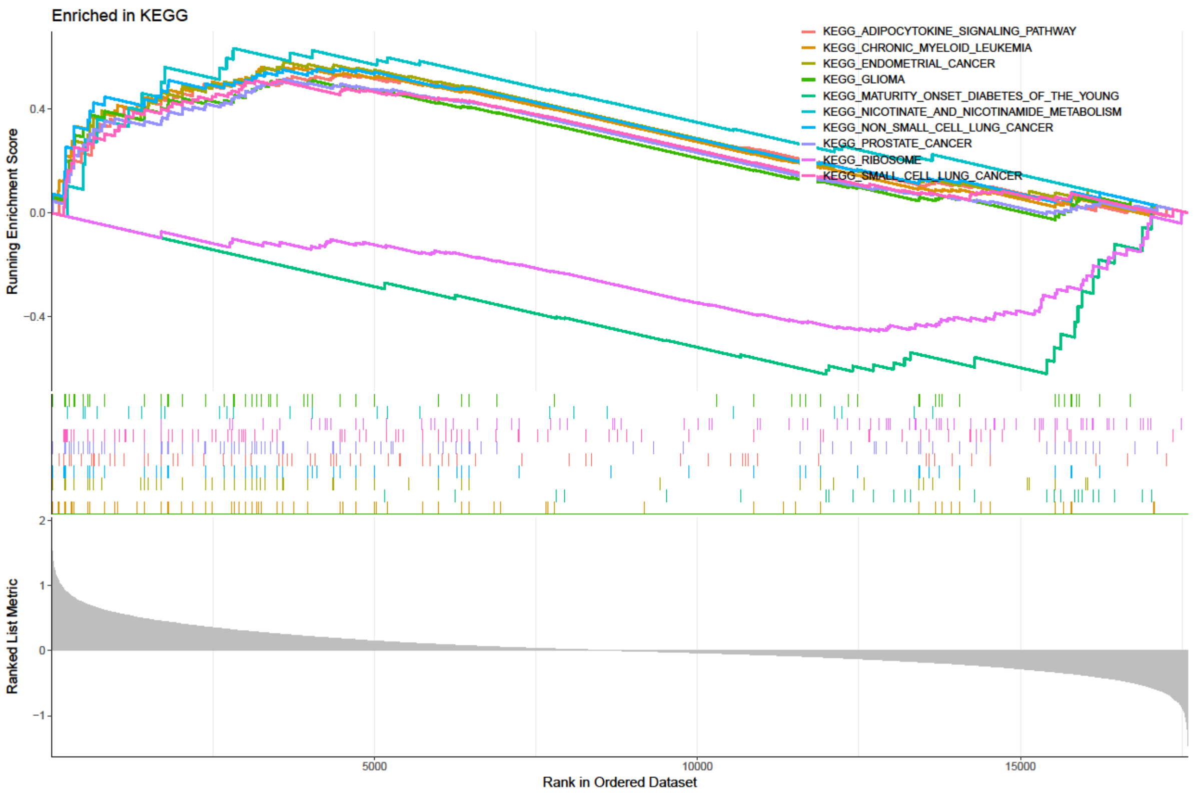

Figure S7. Top 10 pathways ranked by the absolute value of the enrichment score for WRN. The illustration of the figure is the same as the illustration of Figure S4.

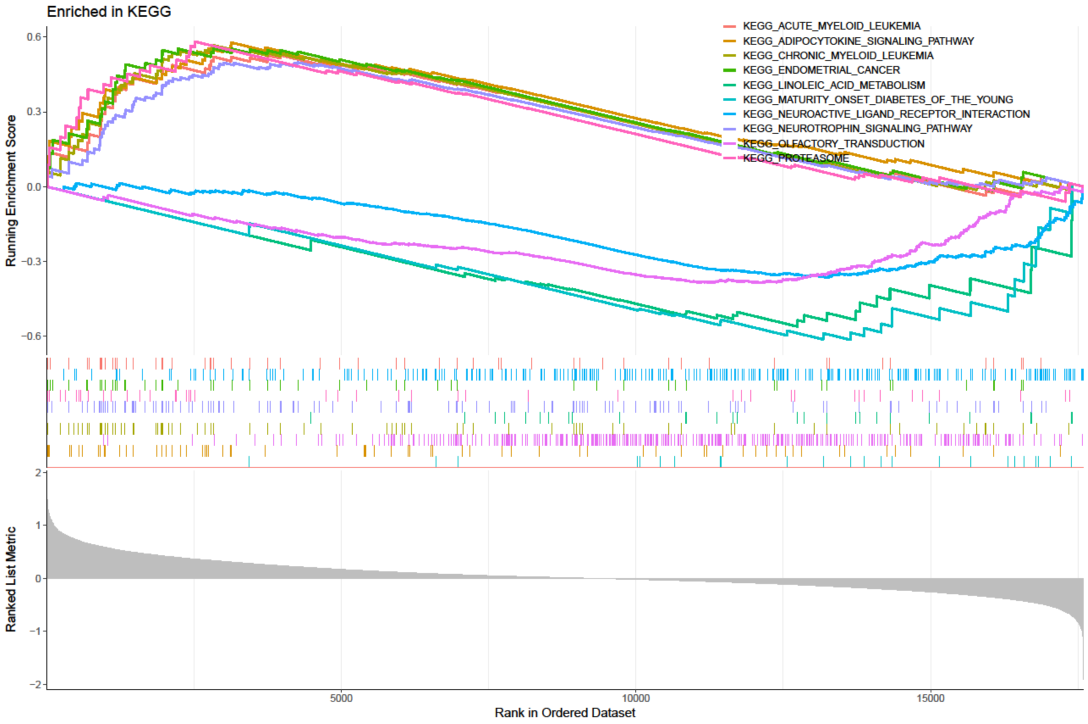

Figure S8. Regulatory network linking feature genes and transcriptional factors. The purple rhombuses indicate feature genes while the blue polygons indicate transcriptional factors.

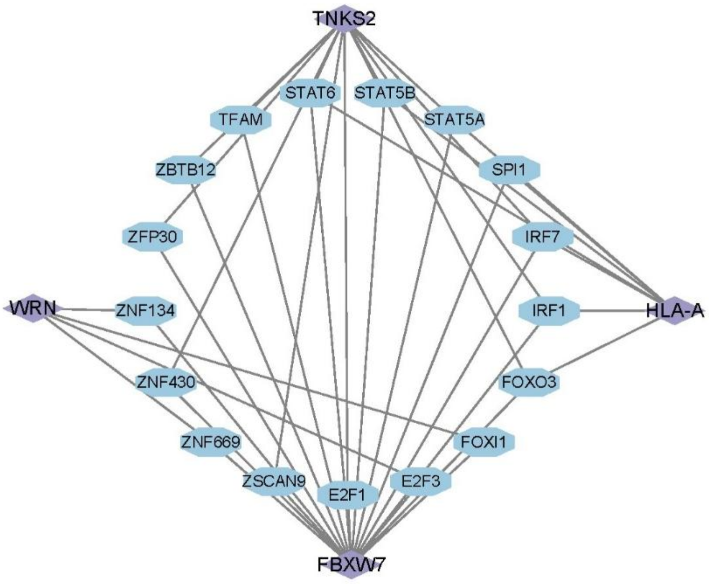

Figure S9. The regulatory network between mRNA and miRNA. The purple rhombuses represent feature genes, and the green ovals represent miRNA.

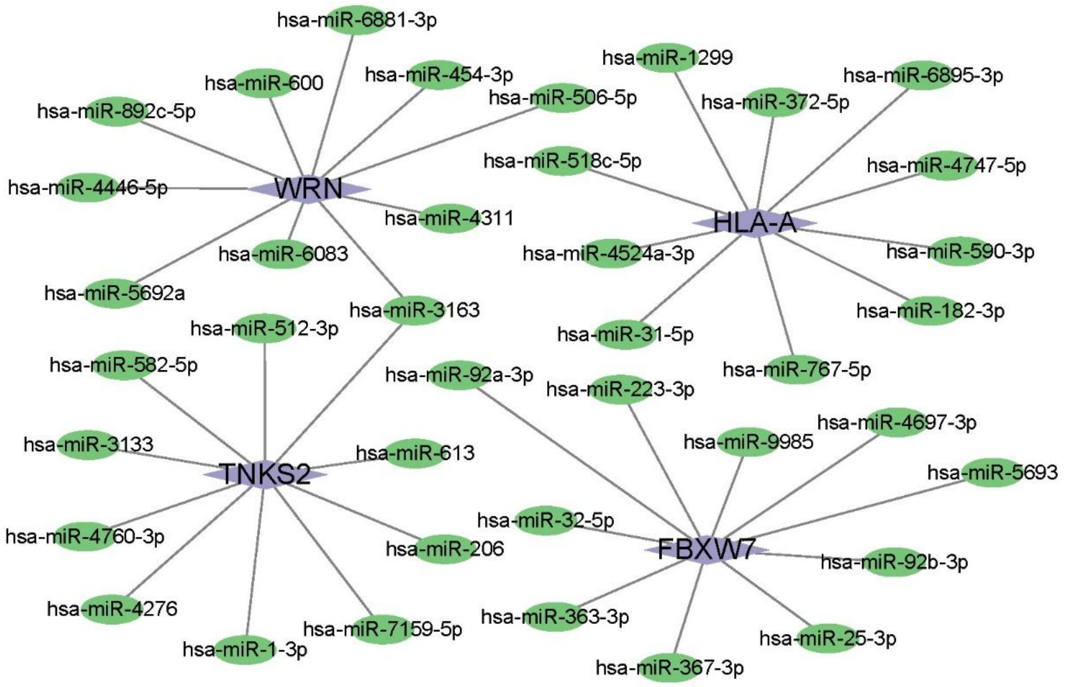

Figure S10. GeneMANIA database gene interacting network.

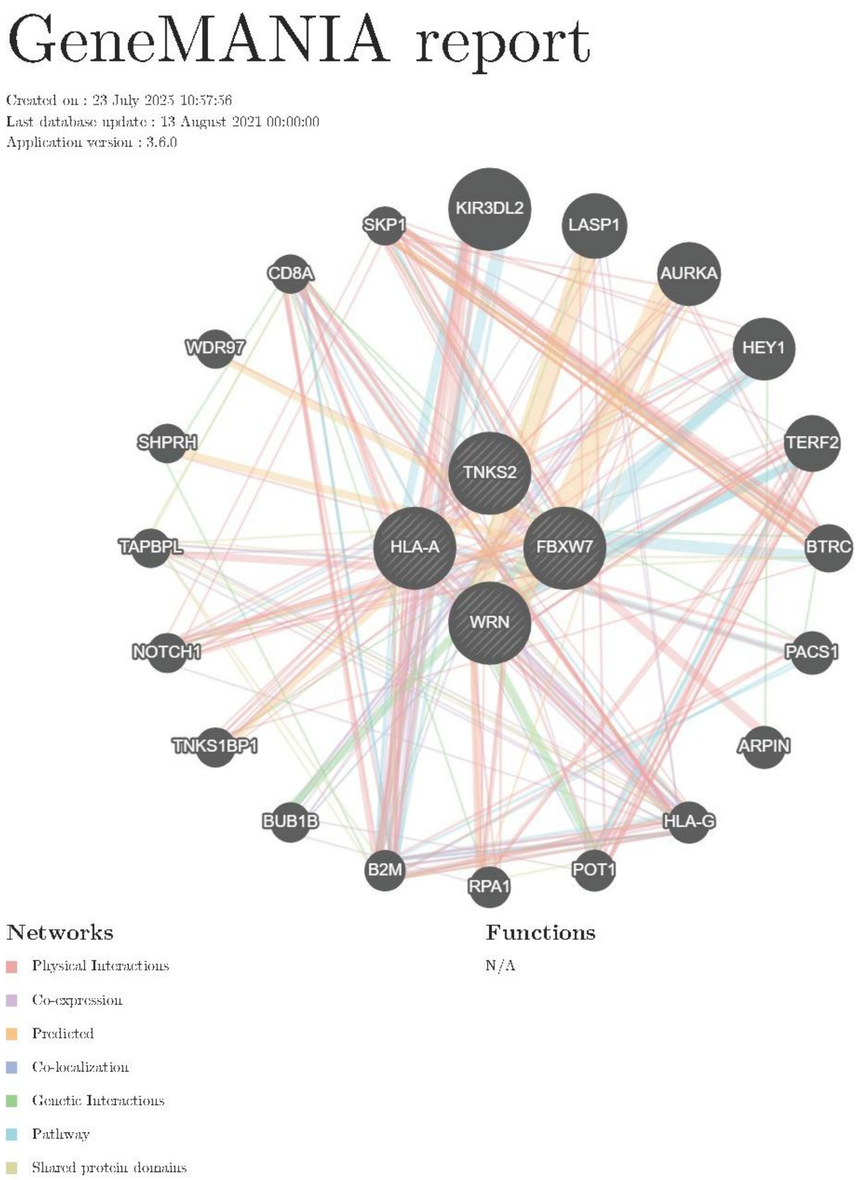

Figure S11. Visualization of molecule docking results of WRN- BERBERRUBINE, TNKS2-2X-121, and HLA-A- BACAMPICILLIN. The left panel of the molecular docking diagram depicts the protein structure in blue ribbon representation, while the small molecule drug is shown in pink. The right panel displays a two-dimensional schematic of the docking interaction results.

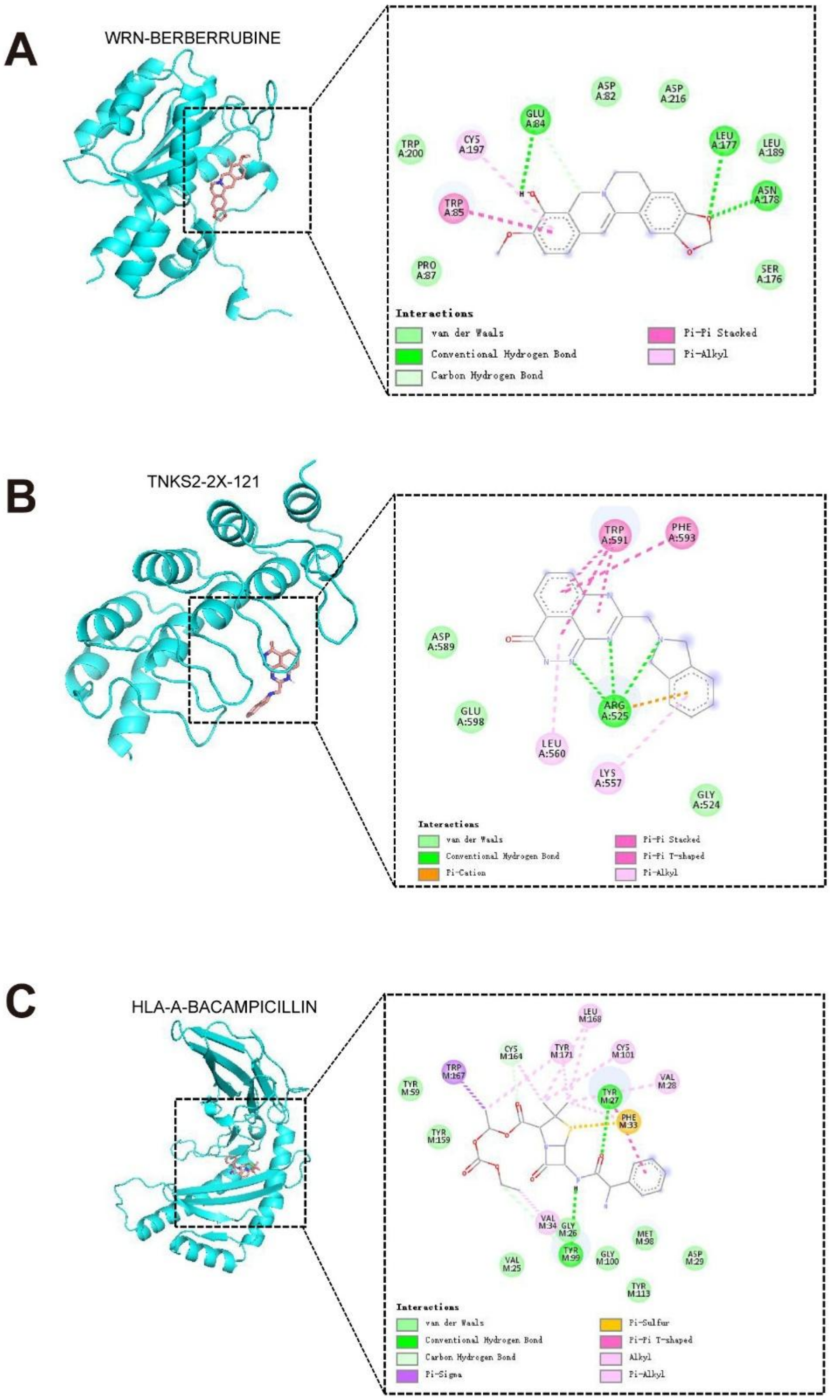

Figure S12. Validation of FBXW7 siRNA transfection efficiency.

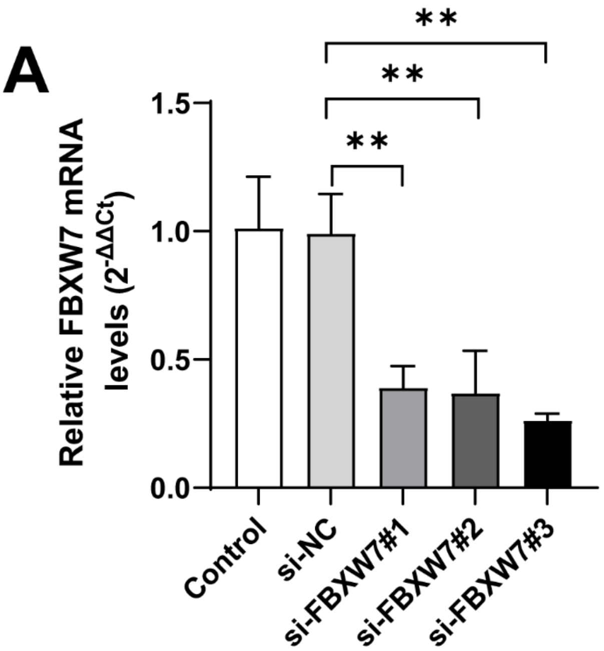

Quantitative real-time PCR (qRT-PCR) analysis showing the relative mRNA expression levels of **FBXW7** in human brain vascular smooth muscle cells (HBVSMCs) after transfection with **si-NC** or three different **si-FBXW7** oligonucleotides. Data are expressed as **mean ± SD** from **three independent experiments (n = 3)**. A significant reduction in FBXW7 mRNA expression was observed in the **si-FBXW7#1, si-FBXW7#2, and si-FBXW7#3** groups compared with the **control** and **si-NC** groups, confirming successful knockdown efficiency. Statistical analysis was performed using one-way ANOVA followed by Tukey's post hoc test; **P < 0.01** (**)

The original EdU assay images for the si-FBXW7 transfection experiment are shown below.

Group: si-FBXW7

Set1

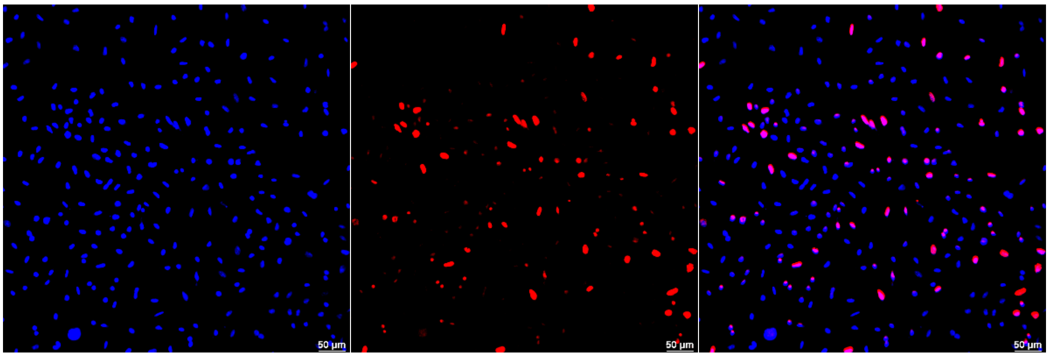

Set2

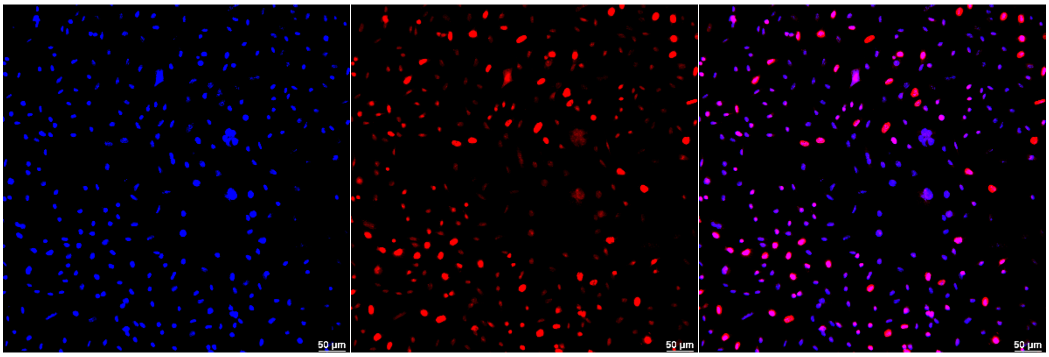

Set3

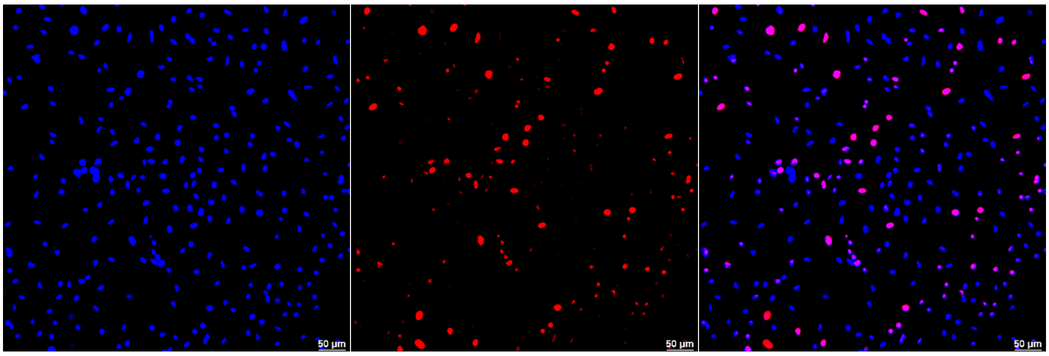

Group: si-NC

Set1

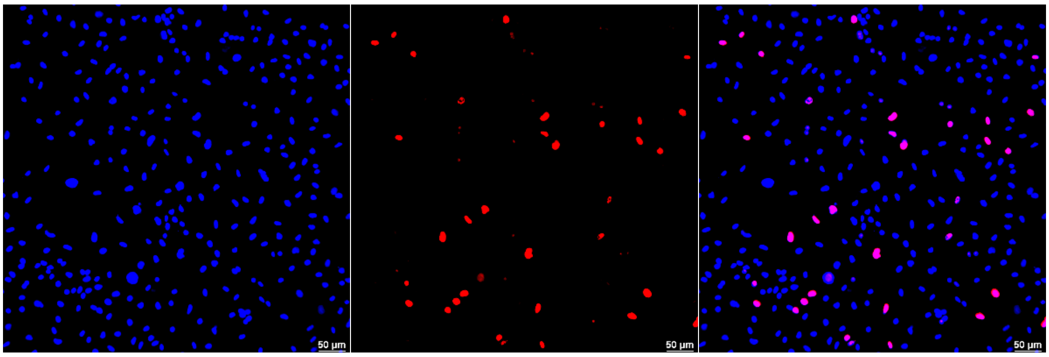

Set2

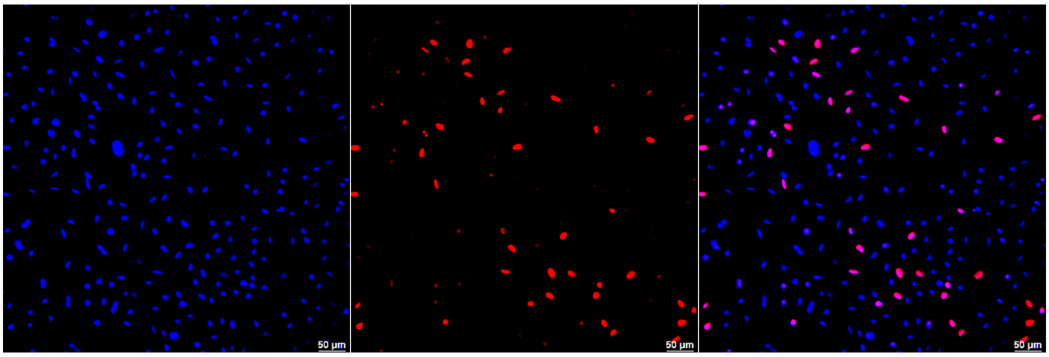

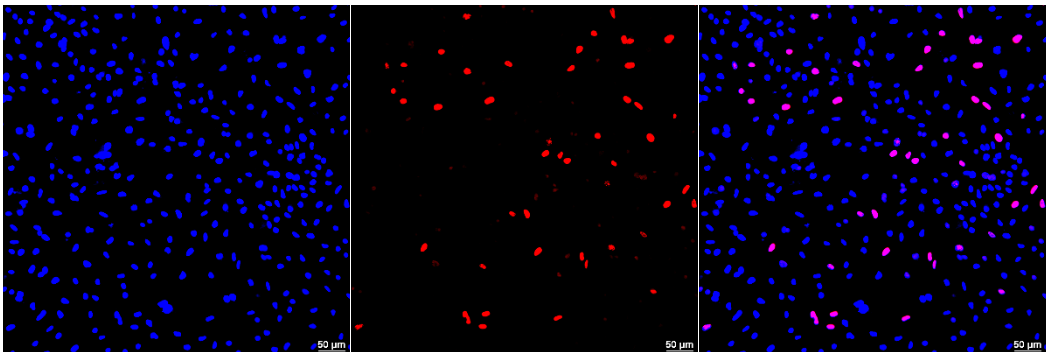

The original scratch wound assay assay images shown below.

Group: si-FBXW7

0 H

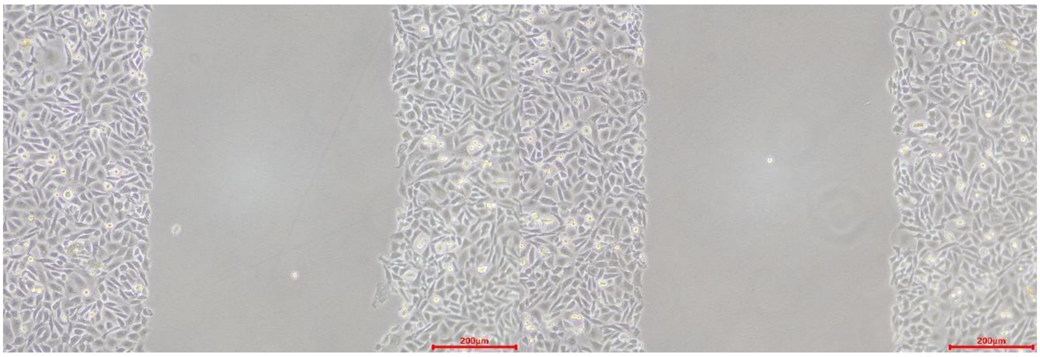

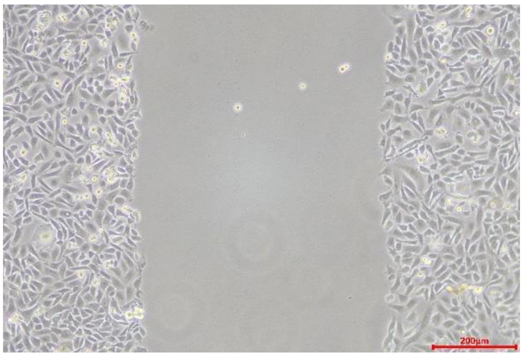

48 H

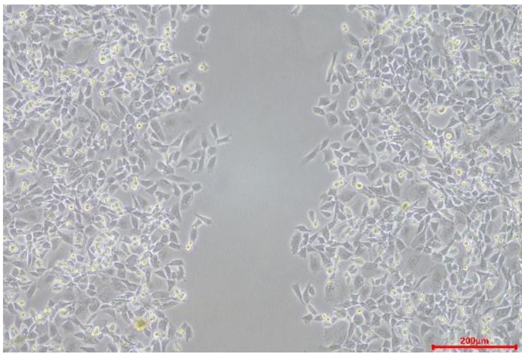

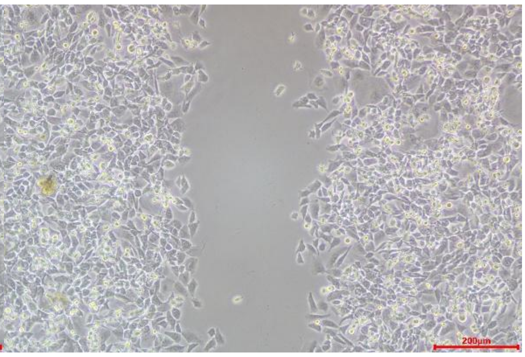

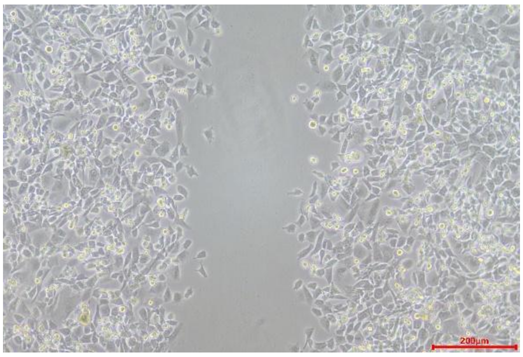

Group: si-NC:

0 H

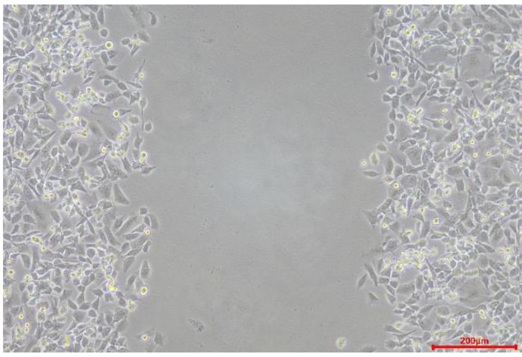

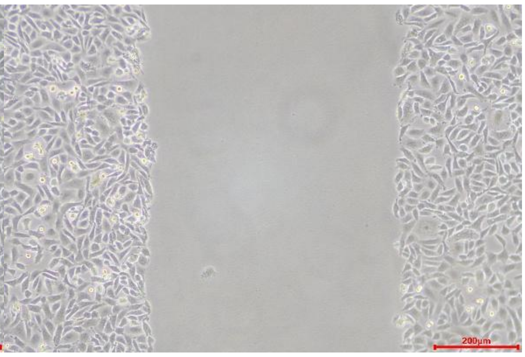

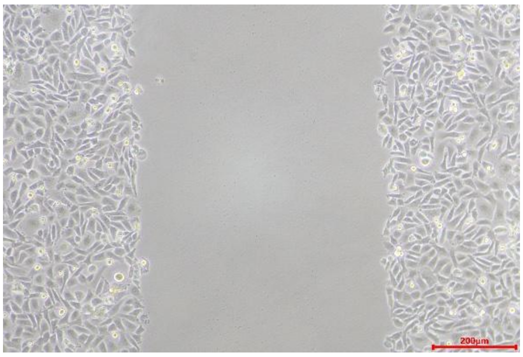

48 H

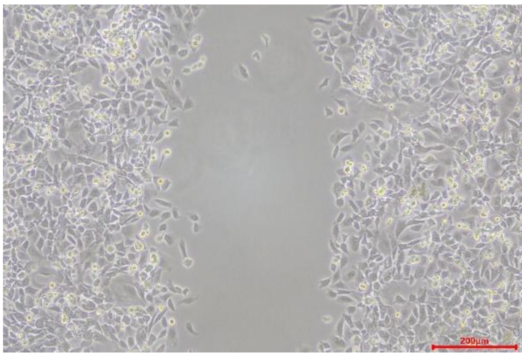
200μm
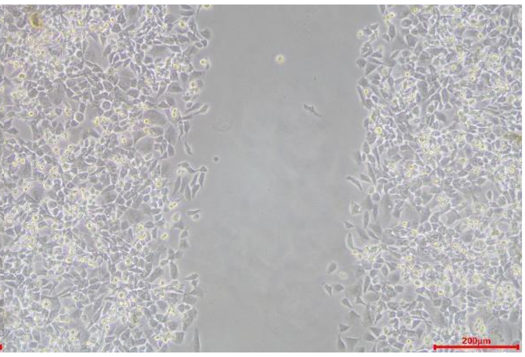
200μm
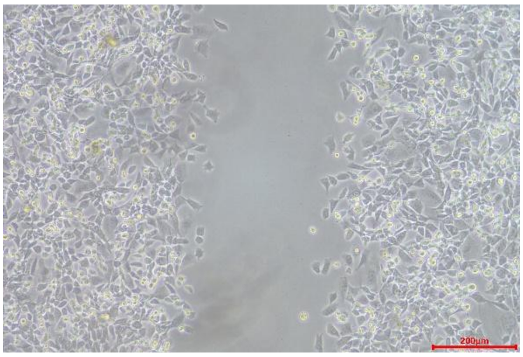
200μm
